# Magnetoreceptor Promotes Hepatocellular Carcinoma Development and Blunts Sorafenib‐Induced Ferroptosis by Regulating Iron Homeostasis

**DOI:** 10.1002/advs.78015

**Published:** 2026-09-27

**Authors:** Tao Wang, Chenghua Song, Xin He, Mengzhou Wang, Wuming Liu, Xiaolin Wang, Yuanyuan Zhang, Jia Zhang, Lin Zhang, Dan Li, Yu Zhang, Zheng Wu, Yi Lyu, Rongqian Wu

**Affiliations:** ^1^ National Local Joint Engineering Research Center for Precision Surgery and Regenerative Medicine Shaanxi Provincial Center for Regenerative Medicine and Surgical Engineering The First Affiliated Hospital of Xi'an Jiaotong University Xi'an Shaanxi China; ^2^ Department of Hepatobiliary and Pancreas Surgery Sichuan Provincial People's Hospital University of Electronic Science and Technology of China Chengdu Sichuan China; ^3^ Department of Hepatobiliary Surgery The First Affiliated Hospital of Xi'an Jiaotong University Xi'an Shaanxi China; ^4^ Department of Gastroenterology The First Affiliated Hospital of Xi'an Jiaotong University Xi'an Shaanxi China; ^5^ Department of Pathology The First Affiliated Hospital of Xi'an Jiaotong University Xi'an Shaanxi China; ^6^ Department of Pediatrics The First Affiliated Hospital of Xi'an Jiaotong University Xi'an Shaanxi China; ^7^ Department of Gastroenterology The Second Affiliated Hospital of Xi'an Jiaotong University Xi'an Shaanxi China

**Keywords:** hepatocellular carcinoma, iron‐sulfur cluster assembly protein, magnetoreceptor, ferroptosis, sorafenib

## Abstract

Iron homeostasis plays a critical role in the pathogenesis and therapeutic management of hepatocellular carcinoma (HCC). Magnetoreceptor (MagR), also known as iron‐sulfur cluster assembly protein 1 (ISCA1), is a conserved protein maintaining iron homeostasis, plays an undefined role in HCC. The present study aimed to elucidate MagR's functional involvement in HCC pathogenesis and sorafenib‐induced ferroptosis, and to explore its therapeutic potential for HCC. Pan‐cancer analyses showed that MagR was significantly upregulated in HCC tissues compared with normal liver tissues, accompanied with advanced stage and poor prognosis in patients. Functional experiments further demonstrated that MagR deficiency suppressed HCC cell proliferation, migration, and invasion while enhancing the sensitivity of these cells to sorafenib‐induced ferroptosis, whereas overexpression of MagR had opposite effects. Mechanistically, MagR promoted HCC progression by sustaining cellular iron homeostasis and preserving mitochondrial function, and mediated ferroptosis sensitivity of HCC cells via regulating intracellular iron accumulation. Notably, in vivo studies showed that MagR ablation not only attenuated the growth of HCC xenograft tumors but also potentiated the antitumor efficacy of sorafenib. Collectively, these findings identify MagR as a potential therapeutic target for HCC and establish that therapeutic strategies targeting MagR could optimize sorafenib‐based treatment regimens for this disease.

## Graphical abstract

Magnetoreceptor (MagR) regulates iron homeostasis and mitochondrial function in hepatocellular carcinoma (HCC) cells. Specifically, MagR deficiency suppresses tumor cell proliferation, migration, and invasion and enhances sorafenib‐induced ferroptosis through iron accumulation. Restoring iron homeostasis with ferritin heavy chain 1 (FTH1) attenuates these ferroptosis‐related and sorafenib‐sensitizing effects. A tumor microenvironment‐responsive nanoparticle combining MagR silencing with sorafenib delivery exerts potent anti‐HCC effects.

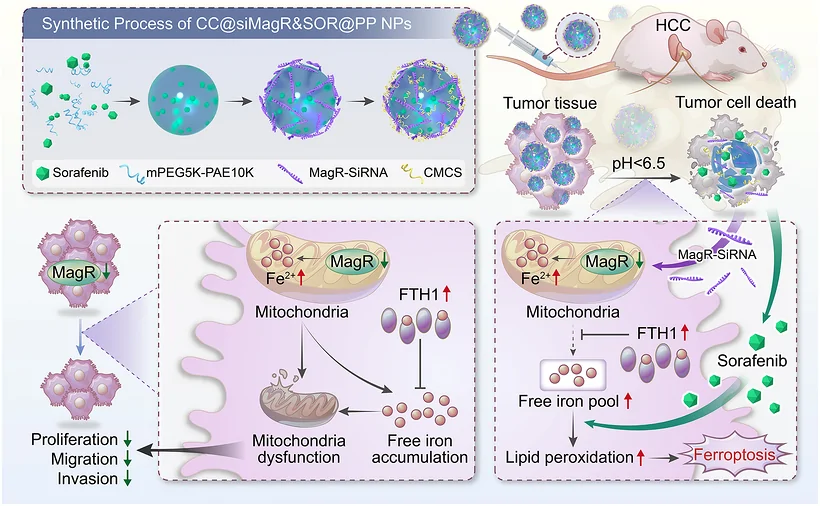

## 1 Introduction

Liver cancer stands as the sixth most common malignancy globally and the third leading cause of cancer‐related death [1, 2]. Hepatocellular carcinoma (HCC) constitutes approximately 90% of primary malignant liver tumors [3, 4]. Although recent advances in surveillance, early detection, diagnosis, and treatment have helped curb HCC development and overall mortality, its incidence and death rates remain unacceptably high [5, 6]. HCC treatment options include hepatectomy, liver transplantation, ablation, and systemic chemotherapy [7, 8]. Surgical interventions work well for early‐stage HCC, but most HCC patients are diagnosed at an advanced stage, leaving systemic therapy as their main option [9, 10]. Unfortunately, current systemic treatments offer limited improvements in overall survival, creating an urgent need to deepen understanding of HCC pathogenesis and identify new diagnostic targets and therapeutic strategies.

Ferroptosis, first described in 2012, is an iron‐dependent form of regulated cell death triggered by lethal lipid peroxide accumulation in cellular membranes [11]. It has been implicated in cancer treatment, neurodegenerative diseases, and ischemia‐reperfusion injury [12, 13]. Unlike other programmed cell deaths (e.g., apoptosis, necroptosis, pyroptosis), ferroptosis exhibits unique morphological and mechanistic features [14]. Its initiation stems from an imbalance between pro‐ferroptotic drivers and cellular defense mechanisms, resulting in the toxic accumulation of oxidized phospholipids [15]. Pro‐ferroptotic processes include metabolic pathways that generate lipid peroxides, such as polyunsaturated fatty acid‐phospholipid biosynthesis, redox‐active iron metabolism, and mitochondrial bioenergetics [16, 17, 18]. Cellular defenses, meanwhile, involve membrane remodeling and antioxidant systems that directly neutralize lipid peroxides [19, 20, 21, 22]. Sorafenib, a first‐line systemic therapy for selected patients with advanced HCC, can act as a ferroptosis inducer via the glutathione peroxidase 4 (GPX4)‐dependent pathway, suppressing solute carrier family 7‐member 11 (SLC7A11) function [23, 24]. Numerous genetic alterations have been shown to enhance or inhibit sorafenib‐induced ferroptosis in cancer cells by regulating antioxidant defenses and/or pro‐ferroptotic processes [25]. These findings have advanced understanding of sorafenib's clinical use, and further research in this area is expected to address critical clinical challenges, such as HCC drug resistance and adverse events associated with sorafenib.

The type A iron‐sulfur cluster assembly protein 1 (ISCA1), also known as the magnetoreceptor (MagR), owing to its capacity to respond to magnetic fields, is a highly conserved protein [26, 27, 28]. It localizes to the mitochondria of most eukaryotes, where it plays a pivotal role in sustaining iron homeostasis [29]. Pathogenic ISCA1 variants are associated with a distinct form of mitochondrial disorder, specifically designated as ISCA1‐related mitochondrial multiple dysfunction syndrome [30]. Functional investigations using animal models have further underscored the biological significance of ISCA1. For example, murine models with neuron‐specific ISCA1 knockout displayed marked reductions in the expression of electron transport chain complex proteins, which was accompanied by a corresponding drop in ATP synthesis, reflecting impaired mitochondrial energy production [31]. In a separate study, Ling et al. developed a novel rat model of dilated cardiomyopathy by specifically knocking down ISCA1 in rat cardiomyocytes [32]. This model exhibited impaired enzyme activity of mitochondrial respiratory chain complexes I, II, and IV, as well as reduced adenosine triphosphate (ATP) production [32]. Notably, the loss of ISCA1 in rat cardiomyocytes also disrupted iron homeostasis, highlighting its dual role in mitochondrial function and iron regulation [29]. Beyond its contributions to mitochondrial physiology and iron metabolism, ISCA1 has clinical relevance as a cancer‐specific prognostic biomarker. It has been identified as such in multiple malignancies, including bladder cancer, clear cell renal carcinoma, and thyroid carcinoma [33, 34, 35].

Despite this expanding body of research, the specific role of MagR (i.e., ISCA1) in the pathogenesis of HCC, as well as its regulatory function in sorafenib‐induced ferroptosis, remains unclear. The aim of this study, therefore, was to clarify the functional role of MagR in the pathogenesis of HCC and sorafenib‐induced ferroptosis, with a concurrent exploration of its relevance to therapeutic strategies.

## 2 Materials and Methods

### 2.1 Patient Samples and Bioinformatic Analyses

The mRNA expression data and clinical information were obtained from The Cancer Genome Atlas (TCGA) database (https://genome‐cancer.ucsc.edu/). This dataset included 375 cases of HCC patients and 50 adjacent non‐tumor tissues. Additional mRNA expression data of primary liver tumor tissues and adjacent non‐tumor tissues were obtained from the Gene Expression Omnibus (GEO) database (https://www.ncbi.nlm.nih.gov/geo/). In this study, 20 pairs HCC tissues and corresponding adjacent normal tissue specimens were obtained from the Biobank of the First Affiliated Hospital of Xi'an Jiaotong University. Immunohistochemistry was used to detect the expression of MagR in HCC tissues and normal liver tissues. Patients and their families signed informed consent voluntarily. This study was approved by the Ethics Committee of the First Affiliated Hospital of Xi'an Jiaotong University.

### 2.2 Antibodies and Reagents

The primary antibody against MagR was purchased from NOVUS. The primary antibodies against peroxisome proliferator‐activated receptor gamma coactivator 1‐α (PGC‐1α), mitofusin 2 (MFN2), dynamin‐related protein 1 (DRP1), transferrin receptor 1 (TFR1), iron regulatory protein 2 (IRP2), Ki‐67, 4‐hydroxynonenal (4‐HNE) and GPX4 were obtained from Abcam. The primary antibodies against mitochondrial transcription factor A (TFAM), SLC7A11, and β‐actin were purchased from Cell Signaling. The primary antibodies against iron regulatory protein 1 (IRP1), ferritin light chain (FTL), and ferritin heavy chain 1 (FTH1) were obtained from Affinity. The Cell Counting kit‐8 (CCK‐8) assay Kit, Mito‐FerroGreen, and FerroOrange were obtained from DOJINDO. ATP, glutathione (GSH), malondialdehyde (MDA), and lactate dehydrogenase (LDH) assay Kit were purchased from Nanjing Jiancheng. Erastin, sorafenib, ferrostatin‐1, Z‐VAD‐FMK, and necrostatin‐1 were obtained from Selleck Chemicals. C11‐BODIPY (581/591), MitoTracker, MitoSOX, and JC‐1 were obtained from Thermo Fisher Scientific. Calcein/PI, the BeyoClick EdU Cell Proliferation Kit and the Ferric and Ferrous Ion Assay Kit were purchased from Beyotime.

### 2.3 Cell Culture

Human HCC cell lines HepG2 (Research Resource Identifier (RRID: CVCL_0027) and Huh7 (RRID: CVCL_0336) were obtained from iCell Bioscience Inc. Both cell lines were authenticated by short tandem repeat (STR) profiling and tested negative for mycoplasma contamination before use. Cells were cultured in Dulbecco's modified Eagle medium (DMEM) medium supplemented with 10% fetal bovine serum and 1% penicillin‐streptomycin mixture and maintained in a humidified atmosphere of 5% CO_2_ at 37°C.

### 2.4 Establishment of Sorafenib‐Resistant Huh7 Cells

Sorafenib‐resistant (SR) Huh7 cells were generated from parental Huh7 cells by continuous exposure to stepwise‐increasing concentrations of sorafenib. Cells were initially treated with 0.1 µM sorafenib, and the concentration was increased by 0.25, 0.5, 1.0, 2.0, 4.0, 8.0, and 10 µM after the cells had recovered stable proliferation. Huh7‐SR cells were subsequently maintained in medium containing 10 µm sorafenib. Resistance was confirmed by comparing sorafenib dose‐response curves and half‐maximal inhibitory concentration (IC_50_) values between parental and sorafenib‐resistant Huh7 cells using the CCK‐8 assay.

### 2.5 Lentivirus and Transfection

The MagR‐overexpressing lentiviral, MagR‐targeting small hairpin RNA (shRNA), and control lentiviral vectors were constructed by Shanghai GeneChem Co., Ltd. To generate stable MagR overexpression and knockdown cell lines, HepG2 and Huh7 cells were transduced with lentivirus for 24 h and then selected with puromycin for at least 7 days.

### 2.6 Plasmids

FTH1 cDNA clone (NM_002032) in GV657 vector was designed by GenePharma. The plasmid deoxyribonucleic acid (DNA) was transfected to Huh7 cells using Lipofectamine 3000 reagent (Thermo Fisher Scientific) following the manufacturer's instructions.

### 2.7 Colony Formation Assay

Cells were seeded into a six‐well plate at a density of 3 × 10^3^ cells/well. After incubation at 37°C for 2 weeks, the colonies were washed twice, fixed with 4% paraformaldehyde for 15 min, and stained with 0.1% crystal violet for 30 min. The stained colony numbers were counted with ImageJ software.

### 2.8 Transwell Assay

Cells were seeded into the top chamber of the transwell plate (Corning) at a density of 4 × 10^4^ cells/well in 200 µL serum‐free DMEM medium with (invasion) or without (migration) Matrigel (Thermofisher). 600 µL DMEM medium supplemented with 20% fetal bovine serum was placed in the lower chamber. After 24 h, the transwells were fixed with 4% paraformaldehyde and stained with 0.1% crystal violet. Then, the remaining cells on the top chamber were removed using a cotton swab, and the migrated cells were photographed by an inverted microscope.

### 2.9 Wound Healing Assay

Cells were seeded into a six‐well plate at a density of 1 × 10^6^ cells/well. After incubation at 37°C for 24 h, a scratch line was performed in the cell monolayer. The cells were then cultured in serum‐free DMEM medium. Images were obtained with an optical microscope and wound width was measured at 0 h and 24 h after the scrape.

### 2.10 EdU Assay

The Huh7 and HepG2 cells proliferation ability was measured by BeyoClick EdU Cell Proliferation Kit according to the instructions. In brief, cells were incubated in medium containing 20 µM EdU for 2 h, then the cells were immobilized, rinsed and permeated sequentially for further staining. Cells were stained with Click Additive Solution for 30 min at room temperature without light. After counterstained with DAPI, the cells were observed with fluorescence microscopy.

### 2.11 Cell Viability Assay

To examine the proliferative ability of HCC cells, cells were seeded at a density of 3 × 10^3^ cells/well in 100 µL DMEM medium in 96‐well plate. From the second day after plating, cell viability was measured at 24 and 48 h using the CCK‐8 assay Kit according to the manufacturer's instructions.

To examine the effects of erastin and sorafenib on HCC cells, cells were seeded at a density of 5 × 10^3^ cells/well in 100 µL DMEM medium in 96‐well plate. Different concentrations of erastin (0, 1.25, 2.5, 5, 10, or 20 µM) or sorafenib (0, 1.25, 2.5, 5, or 10 µM) was added to the 96‐well plate. After 24 h, 10 µL of CCK‐8 reagent was added for further incubation at 37°C for 2 h. The CCK‐8 results were measured at 450 nm using a microplate reader.

### 2.12 Western Blotting

The total proteins of cells were extracted using RIPA buffer containing cocktail of protease inhibitor and phosphatase inhibitor cocktails (Beyotime). Protein concentrations were determined using a bicinchoninic acid (BCA) protein assay kit (Nanjing Jiancheng). Western blotting was performed as previously described. Briefly, the proteins were separated by SDS‐PAGE (Bio‐Rad) and then transferred onto poly (vinylidene fluoride) membranes (Bio‐Rad). After blocking with 5% skim milk for 1 h at room temperature, the membranes were incubated with primary antibodies at 4°C overnight and then with secondary antibodies at room temperature for 1 h. The protein expression was visualized using a chemiluminescence system (Bio‐Rad) and analyzed by ImageJ software.

### 2.13 Quantitative Real‐Time

Total RNA extraction, reverse transcription and quantitative real‐time ploymerase chain reaction (qRT‐PCR) were carried out as previously described. Briefly, total RNA was extracted from cells using TRIzol (Takara), and then cDNA was synthesized with PrimeScript RT Master Mix (Perfect Real Time). Subsequently, the cDNA was amplified by qRT‐PCR using SYBR Green PCR Master Mix (Takara). The following primer sets (Takara) were used: Human MagR (forward, 5′ TCCTTAGTCCGGGCAACTG 3′; reverse, 5′ CCTGGTTCGGACACCAACTT 3′). TFR1 (forward, 5′ TGAAGGTCTGACACGTCTGCCTA 3′; reverse, 5′ TTCACTCACGGAGCTTCGAACTTA 3′). IRP1 (forward, 5′ GGGTTATGTAGGAGCAATGCAGA 3′; reverse, 5′ GAACACAAACTGGCTGGGTGTTA 3′). IRP2 (forward, 5′ TCTCCATGCTGACATTTGAAAG 3′; reverse, 5′ TGAAAGCAAATGCACACGAC 3′). FTL (forward, 5′ GCTACGAGCGTCTCCTGAAGATG 3′; reverse, 5′ CCAGGGCATGAAGATCCAAA 3′). FTH1 (forward, 5′ AGCTCTACGCCTCCTACGTT 3′; reverse, 5′ GCAGCTTCATCAGTTTCTCAGCAT 3′). GAPDH (forward, 5′ CCAGGTGGTCTCCTCTGA 3′; reverse, 5′ GCTGTAGCCAAATCGTTGT 3′) served as an internal control. Relative gene expression was calculated using the 2^− ΔΔCt^ method.

### 2.14 Calcein/PI Staining

Cells were seeded into a 12‐well plate at a density of 1 × 10^5^ cells/well and treated with the indicated concentration of erastin or sorafenib for 24 h. Calcein/PI solution was then added to each well and incubated for 15 min in the dark. Images were obtained with fluorescence microscopy.

### 2.15 Lipid Peroxidation

The lipid peroxidation of cells was detected by C11‐BODIPY (581/591). Cells were seeded into a six‐well plate at a density of 2 × 10^5^ cells/well. After erastin or sorafenib treatment, cells were cultured in serum‐free DMEM medium containing 10 µm C11‐BODIPY for 30 min. The cells were resuspended in phosphate‐buffered saline (PBS) for flow cytometry analysis.

### 2.16 Confocal Microscopy

Cells were seeded into confocal dishes at a density of 1 × 10^4^ cells/well. Then the cells were stained with MitoTracker (100 nM), Mito‐FerroGreen (1 µM), FerroOrange (1 µM), or MitoSOX (5 µM) respectively according to manufacturer's instructions. Images were obtained using a confocal fluorescence microscope.

### 2.17 Mitochondrial Membrane Potential

The mitochondrial membrane potential (MMP) of HCC cells was measured using the JC‐1 mitochondrial potential sensor. The HCC cells were stained with the JC‐1 probe reagent (10 µg/mL) for 30 min as recommended by the manufacturer. Then Images were captured using a confocal microscope.

### 2.18 Iron Assay

Total iron levels and the ratio of ferrous ions to ferric ions (Fe^2+^/Fe^3+^) in HCC cells were assessed by Ferric and Ferrous Ion Assay Kit according to the instructions provided by the manufacturer.

### 2.19 Transmission Electron Microscopy

Cells were observed using a transmission electron microscope (TEM) by a single electron microscopist after stained with uranyl acetate and lead citrate.

### 2.20 ATP, GSH, LDH, and MDA Detection

The level of intracellular ATP was detected by ATP Assay Kit. The level of intracellular GSH was detected by GSH Assay Kit. The level of intracellular MDA was detected by MDA Assay Kit. The level of LDH of the cellular supernatant was detected by LDH Assay Kit. All the procedures were performed following the manufacturer's instructions.

### 2.21 Mouse Xenograft Models

The mouse xenograft experiment was approved by the Animal Ethics Committee of Xi'an Jiaotong University. 4–5‐week‐old male BALB/c nude mice were used to establish xenograft models. The Huh7 cells were washed and resuspended in PBS at a density of 2.5 × 10^7^ cells/mL. 5 × 10^6^ cells in 200 µL PBS were injected subcutaneously into the flank of the nude mice. Tumor volume was measured every 3 days, and mice were sacrificed for tumor on day 24.

In the sorafenib treatment experiment, nude mice were administrated with sorafenib (10 mg/kg, intraperitoneally, once every other day) at day 7 after Huh7 cells injection for 2 weeks. Tumor volume was measured every 3 days, and the tumors were harvested on day 14 after the first treatment of sorafenib. Tumor volume was determined using the following equation:

$$\mathrm{Volume}=\mathrm{length}\;\times \;{\mathrm{width}}^{2}\;\times \;0.5$$

#### 2.21.1 Hematoxylin and eosin (H&E) Staining

Tumor tissues fixed in paraformaldehyde (4%) for 48 h were embedded in paraffin. 5‐µm‐thick tumor tissue sections were stained with H&E. All the images were obtained using an optical microscope.

### 2.22 Immunohistochemistry Staining

Immunohistochemistry (IHC) staining of tumor was performed as previously described [36] and the pictures were obtained with an optical microscope. The primary antibodies used were listed as follows: Ki‐67, MagR, GPX4, 4‐HNE and FTH1 antibodies.

### 2.23 CC@siMagR&SOR@PP NPs

The materials and methods about the synthesis of CC@siMagR&SOR@PP NPs and their application in treating mouse liver carcinoma in situ were described in the Supporting Information.

### 2.24 Statistical Analysis

All statistical analyses were performed by SPSS software (version 22.0) and GraphPad Prism (version 8.0.1). The results are presented as the mean ± standard deviation (SD). Student's *t*‐test was used to compare the differences between two groups and one‐way ANOVA was used for comparisons among multiple groups. *p* < 0.05 was considered to be statistically significant.

## 3 Results

### 3.1 MagR is Upregulated in HCC and Correlates With Poor Prognosis

**FIGURE 1:**
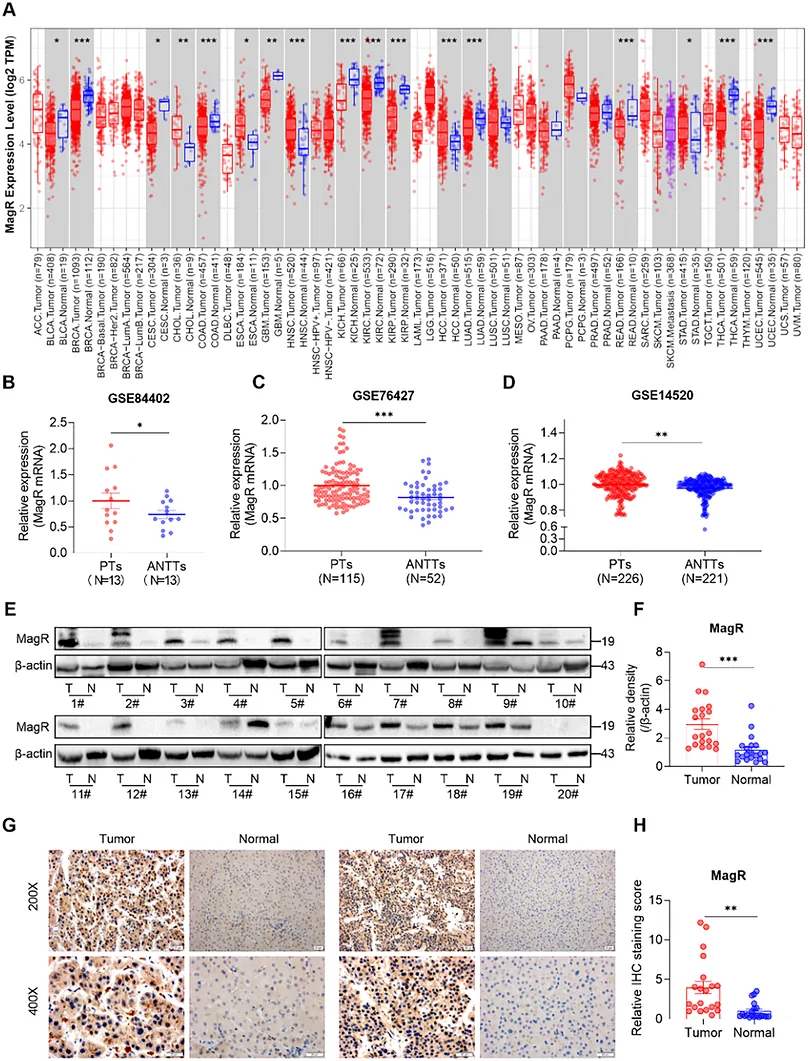
Expression analysis and clinicopathological characteristics of MagR in hepatocellular carcinoma. (A) Expression of MagR in human cancers based on the TCGA database. (B–D) Expression of MagR in primary tumor tissues (PTs) and adjacent non‐tumor tissues (ANTTs) based on GEO database. (E, F) Expression of MagR protein in HCC tissues and adjacent normal tissues (E), with the quantitative analysis (F). (G, H) Representative images of MagR IHC staining in HCC tissue and normal liver tissue (G), with the quantitative analysis (H). HCC, hepatocellular carcinoma; PTs, primary tumor tissues; ANTTs, adjacent non‐tumor tissues. Data are presented as mean ± SD; *p < 0.05, **p < 0.01, and ***p < 0.001).

The expression profile of MagR across various human cancer types was retrieved from the TCGA database and subsequently analyzed using the TIMER2 database. As illustrated in Figure 1A, MagR expression was markedly higher in HCC samples than in non‐cancerous counterparts. This observation suggests that elevated MagR expression may be associated with the initiation or progression of HCC. To validate and extend these findings, we further analyzed MagR expression in HCC using data from the GEO database. As presented in Figure 1B–D, compared with paired adjacent non‐tumor tissues (ANTTs), the expression of MagR was significantly higher in primary tumor tissues (PTs), which was consistent with the TCGA‐derived results. To confirm these transcript‐level observations at the protein level, we examined the expression of MagR protein in 20 paired samples of HCC tumor tissue and adjacent normal tissue. MagR protein levels were substantially increased in HCC tumor tissues than in adjacent normal tissues (Figure 1E,F). We further evaluated MagR protein expression using immunohistochemistry (IHC). Representative IHC images of HCC tissues and adjacent non‐tumor tissues with varying MagR expression levels are displayed in Figure 1G. Notably, high MagR protein expression was predominantly detected in HCC tumor tissues (Figure 1H). Then, we expanded our analysis to a larger cohort, including 375 HCC tissues and 50 adjacent non‐tumor tissues. We next explored the correlation between MagR expression and key clinicopathological features of HCC, including T‐stage, N‐stage and pathologic stage. As shown in Figure S1A, elevated MagR expression was significantly associated with more advanced tumor clinicopathological characteristics (e.g., higher T‐stage, advanced pathologic stage). Kaplan–Meier survival analysis was performed to assess the prognostic value of MagR in HCC. The results demonstrated that MagR expression was positively correlated with poor clinical outcomes, including shorter overall survival, disease‐specific survival, and progression‐free interval ((Figure S1B).

Collectively, these multi‐dimensional analyses confirm that MagR is significantly upregulated in primary HCC tissues compared with normal tissues. Moreover, MagR may serve as a valuable predictive biomarker for both the aggressive clinicopathological features and unfavorable prognosis of HCC.

### 3.2 MagR Deficiency Inhibits, Whereas MagR Overexpression Enhances, HCC Cell Proliferation, Migration, and Invasion

**FIGURE 2:**
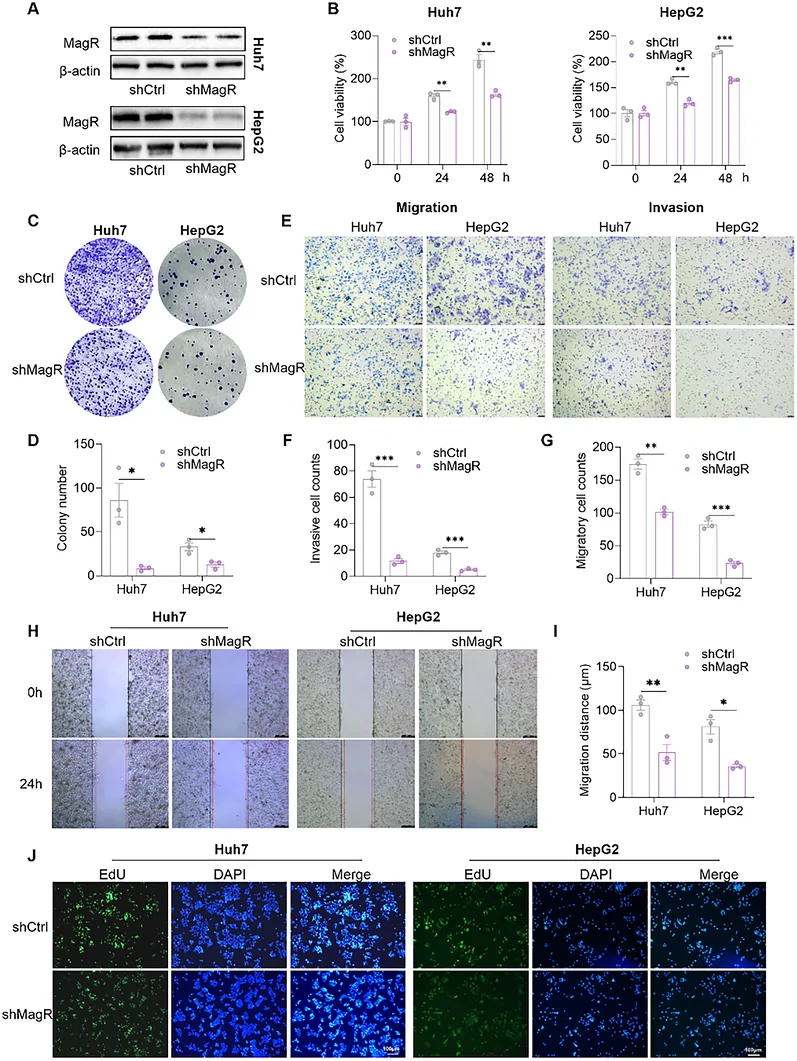
MagR deficiency repressed proliferation, migration, and invasion of HCC cells. All these experiments were conducted on Huh7 and HepG2 cells. (A) The protein expression of MagR in HCC cells transfected with negative control or MagR knockdown constructs (shCtrl and shMagR). (B) The proliferative ability of negative control or MagR‐knockdown HCC cells. (C, D) Representative images of the colony formation (C), with the quantitative analysis (D). (E–G) Representative images of migration and invasion (E), with the quantitative analysis (F, G). (H,I) Representative images of wound‐healing (H), with the quantitative analysis (I). (J) Representative images of EdU staining. *n* = 3; data are presented as mean ± SD; *p < 0.05, **p < 0.01, and ***p < 0.001.

To clarify MagR's functional role in HCC, we first established the MagR‐knockdown models in two HCC cell lines (Huh7 and HepG2) using lentiviral‐mediated gene silencing. As validated in Figure 2A, MagR protein expression was dramatically reduced in the knockdown cells, confirming the success of the gene interference strategy. Functional assays were then performed to evaluate the impact of MagR deficiency on HCC cell behaviors. The CCK‐8 assay revealed that compared with control cells, MagR‐knockdown HCC cells exhibited a significantly impaired proliferative capacity at both 24 and 48 h (Figure 2B). This inhibitory effect on proliferation was further supported by colony formation assays. As illustrated in Figure 2C,D, MagR silencing led to a marked reduction in the number of colonies formed by HCC cells. In addition to proliferation, we assessed the migratory and invasive potentials of MagR‐knockdown cells. Transwell assays demonstrated that MagR deficiency substantially attenuated the migration and invasion capabilities of HCC cells (Figure 2E–G). Specifically, the wound‐healing assay showed that MagR‐knockdown cells had a shorter migratory distance compared with control cells at the indicated time points (Figure 2H,I). EdU staining, which labels proliferating cells, further confirmed the reduced proliferative activity of MagR‐knockdown HCC cells (Figure 2J). To validate the specificity of MagR's role, we complemented these experiments with MagR overexpression in HCC cells. Conversely, MagR overexpression exerted the opposite effect. It significantly enhanced the proliferation, migration, and invasion of HCC cells (Figure S2), further reinforcing MagR's pro‐tumorigenic function.

Collectively, these in vitro functional analyses demonstrate that MagR deficiency represses the proliferation, migration, and invasion of HCC cells, while MagR overexpression promotes these malignant phenotypes, providing direct evidence that MagR plays a critical role in driving the aggressive biological behaviors of HCC cells.

### 3.3 MagR Expression is Associated With Mitochondrial Function and the Accumulation of Free Iron

**FIGURE 3:**
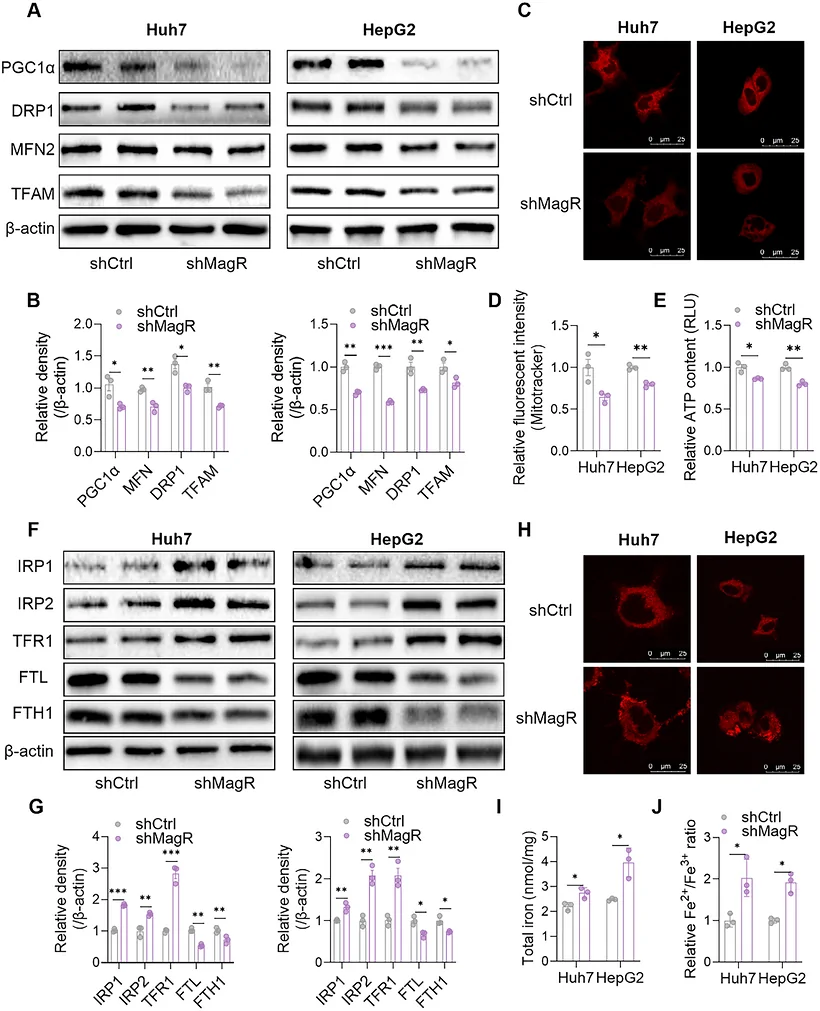
Suppression of MagR induced the dysfunction of mitochondria and accumulation of free iron in HCC cells. All these experiments were conducted on Huh7 and HepG2 cells. (A, B) Protein expression of PGC‐1α, DRP1, MFN2, and TFAM in negative control or MagR‐knockdown HCC cells (A), with the quantitative analysis (B). β‐actin was used as a loading control. (C, D) Representative MitoTracker fluorescence images (C), with the quantitative analysis (D). (E) ATP levels. (F, G) the protein expression of IRP1, IRP2, TFR1, FTL, and FTH1 in negative control or MagR‐knockdown HCC cells (F), with the quantitative analysis (G). β‐actin was used as a loading control. (H)Representative FerroOrange fluorescence images. (I) Total iron levels. (J) Fe^2+^/Fe^3+^ ratio. *n* = 3; data are presented as mean ± SD; *p < 0.05, **p < 0.01, and ***p < 0.001.

MagR is a mitochondrial iron‐sulfur (Fe─S) cluster assembly protein. To investigate whether MagR regulates mitochondrial function and cellular iron homeostasis in HCC cells, we assessed the impact of MagR deficiency on mitochondrial function by examining the expression of key mitochondrial regulatory proteins. As shown in Figure 3A,B, compared with control cells, MagR knockdown HCC cells exhibited significantly reduced protein levels of PGC‐1α (a master regulator of mitochondrial biogenesis), MFN2 (a mediator of mitochondrial fusion), DRP1 (a driver of mitochondrial fission), and TFAM (a core regulator of mitochondrial DNA replication and transcription), suggesting perturbations in mitochondrial biogenesis and dynamics. To further validate mitochondrial dysfunction, we used MitoTracker, a canonical fluorescent probe whose signal intensity correlates with mitochondrial quantity and functional integrity [37]. As illustrated in Figure 3C,D, MagR knockdown cells displayed markedly weaker MitoTracker fluorescence intensity than control cells. Consistently, the ATP level was lower in MagR knockdown cells than control cells (Figure 3E). The mitochondrial reactive oxygen species (ROS) levels in HCC cells were measured using MitoSOX Red reagent. As shown in Figure S3A,B, the mitochondrial ROS level was higher in MagR knockdown cells. JC‐1 dye was used to further investigate the mitochondrial membrane potential (MMP, ΔΨm) in HCC cells. The MMP was decreased in MagR‐knockdown cells than control cells (Figure S3C,D). Taken together, these results confirmed that MagR deficiency impaired mitochondrial function in HCC cells.

Since Fe─S cluster biogenesis is dependent on free iron, we subsequently explored whether MagR regulates cellular Fe^2^^+^ levels. Iron‐responsive proteins 1 and 2 (IRPs) are well‐characterized key regulators of iron homeostasis [38]. Upon activation, IRPs bind to iron‐responsive elements (IREs) in target mRNAs: this interaction accelerates the degradation ofFTH1 (FTH1 is the major iron‐storage protein) while stabilizing TFR1 mRNA (TFR1 mediates iron uptake), ultimately triggering a cellular iron starvation response [39]. As shown in Figure 3F,G, MagR knockdown induced a robust iron starvation response, as evidenced by elevated protein expression of IRP1, IRP2, and TFR1, alongside a concomitant reduction in FTL and FTH1 levels. At the transcriptional level, the mRNA of IRP1, IRP2 and TFR1 was higher, while the mRNA of FTL and FTH1 was lower in MagR‐knockdown cells than control cells (Figure S3E,F). Meanwhile, FerroOrange (a fluorescent probe for detecting labile Fe^2^^+^) staining showed increased fluorescence intensity in MagR‐knockdown cells, indicating that MagR deficiency promoted the accumulation of cellular Fe^2^^+^ (Figure 3H). Consistently, the total iron level and the ratio of Fe^2+^/Fe^3+^ ratio in MagR‐knockdown cells were higher than in control cells (Figure 3I,J).

To confirm the specificity of MagR's role, we performed complementary experiments in MagR‐overexpressing HCC cells. As presented in Figure S4, MagR overexpression exerted the opposite effects: it enhanced mitochondrial function (as reflected by upregulated mitochondrial regulatory proteins, stronger MitoTracker fluorescence, and higher ATP level) and blocked the accumulation of cellular Fe^2^^+^ (as shown by lower total iron level and reduced FerroOrange signal).

Collectively, these findings demonstrate that MagR is required to maintain normal mitochondrial function and cellular iron homeostasis in HCC cells, linking MagR expression to two critical biological processes that support HCC cell survival and malignancy.

### 3.4 FTH1 Overexpression Counteracts the Biological Effects Caused by MagR Suppression

**FIGURE 4:**
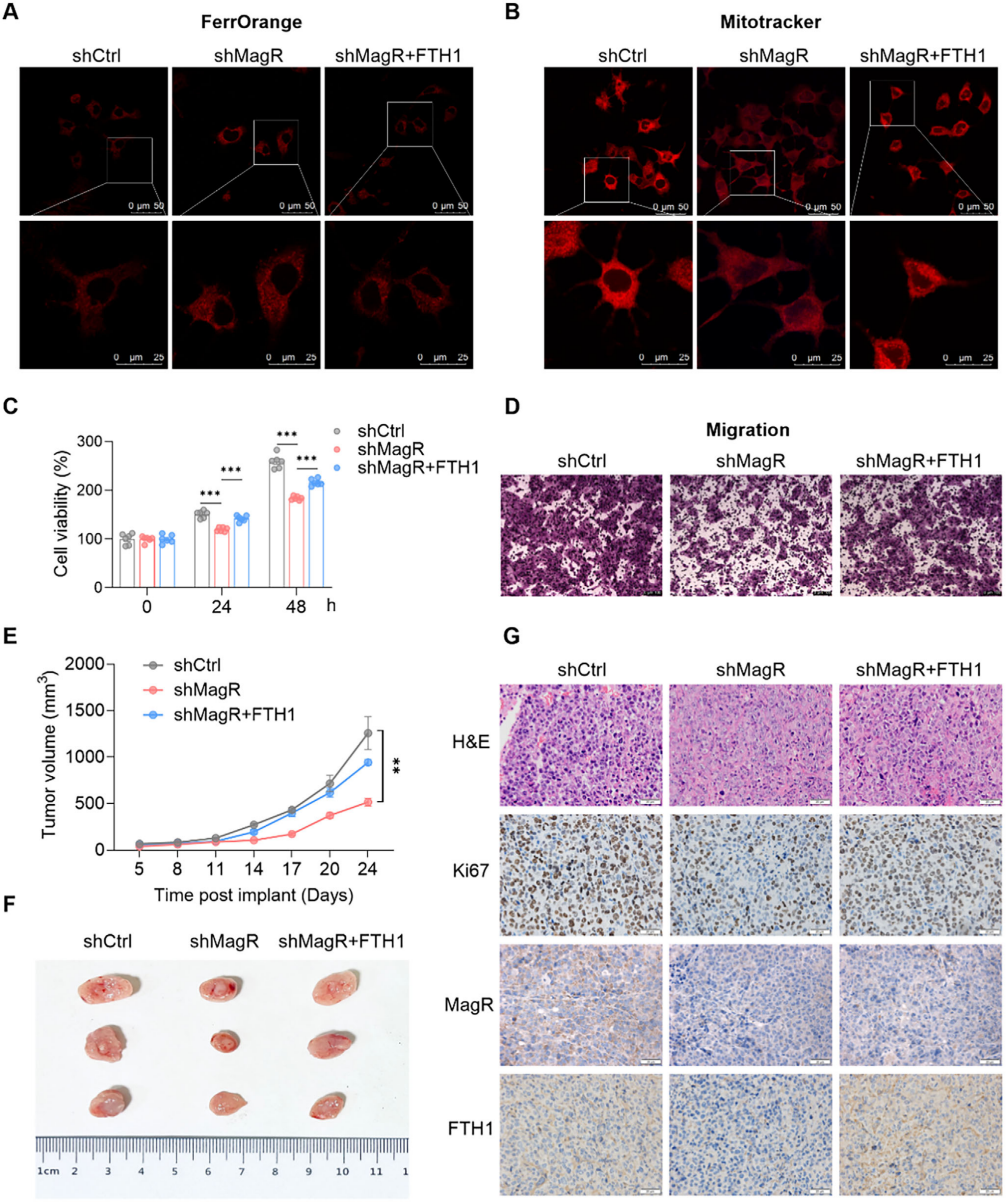
Free iron depletion by FTH1 overexpression counteracted the influence by MagR suppression. All these experiments were conducted on Huh7 cells. (A) Representative FerroOrange fluorescence images of negative control, MagR‐knockdown, and FTH1 overexpression HCC cells. (B) Representative MitoTracker fluorescence images of the indicated groups. (C) The proliferative ability the indicated groups. (D) Representative migration images. (E, F) Tumor volumes of these three groups were measured every 3 days (E), with the representative tumor at the 24th day (F). (G) H&E and IHC analysis for Ki‐67, FTH1, and MagR in indicated tumor specimens. *n* = 3; data are presented as mean ± SD; **p < 0.01 and ***p < 0.001.

Based on the preceding findings, MagR knockdown significantly impairs mitochondrial function and triggers an iron starvation response, ultimately leading to the accumulation of cellular Fe^2^^+^. FTH1 possesses ferroxidase activity, which catalyzes the oxidation of labile Fe^2^^+^ to inactive Fe^3^^+^. Critically, this process enables iron storage in a soluble, non‐toxic form, thereby maintaining cellular iron homeostasis [40]. Given this, we hypothesized that FTH1 overexpression might restore the disrupted iron homeostasis and mitochondrial dysfunction induced by MagR silencing, and in turn, reverse the suppressed malignant biological behaviors of HCC cells. To test this hypothesis, we first transfected an FTH1 overexpression plasmid into MagR‐knockdown Huh7 cells to reconstitute FTH1 expression. As shown in Figure 4A, FTH1 overexpression reduced the FerroOrange fluorescence intensity in MagR‐knockdown Huh7 cells, directly demonstrating that FTH1 overexpression mitigated the Fe^2^^+^ accumulation caused by MagR deficiency. Next, we evaluated whether FTH1 overexpression could rescue mitochondrial function, proliferation, and migration in MagR‐knockdown HCC cells. Consistent with our earlier observations, MagR knockdown significantly decreased MitoTracker fluorescence intensity in Huh7 cells, indicating mitochondrial dysfunction (Figure 4B). However, this decrease was reversed by FTH1 overexpression, confirming that FTH1 could alleviate MagR knockdown‐induced mitochondrial impairment. Additionally, functional assays revealed that FTH1 overexpression abrogated the proliferation arrest of MagR‐knockdown Huh7 cells and restored their migratory capacity (Figure 4C,D).

To validate these in vitro findings in vivo, we performed xenograft tumor experiments. As shown in Figure 4E,F, MagR knockdown markedly suppressed tumor growth, whereas FTH1 overexpression restored tumor growth to levels comparable to those in control group, which was consistent with our in vitro findings. H&E staining and IHC results further supported these observations. MagR‐silenced tumor tissues exhibited extensive necrotic regions, accompanied by nuclear pyknosis and fragmentation, as well as a significant reduction in Ki‐67 (a proliferation marker) expression (Figure 4G). In contrast, FTH1 overexpression not only preserved the normal morphology of tumor cells but also elevated Ki‐67 expression (Figure 4G), confirming its ability to rescue the anti‐tumor effects of MagR knockdown.

In conclusion, these results collectively demonstrate that restoring cellular iron homeostasis via FTH1 overexpression can reverse the inhibitory effects of MagR knockdown on HCC cell function, both in vitro and in vivo, highlighting FTH1 as a key mediator in MagR‐regulated iron metabolism and HCC progression.

### 3.5 MagR Deficiency Enhances Erastin‐Induced Ferroptosis in HCC Cells

**FIGURE 5:**
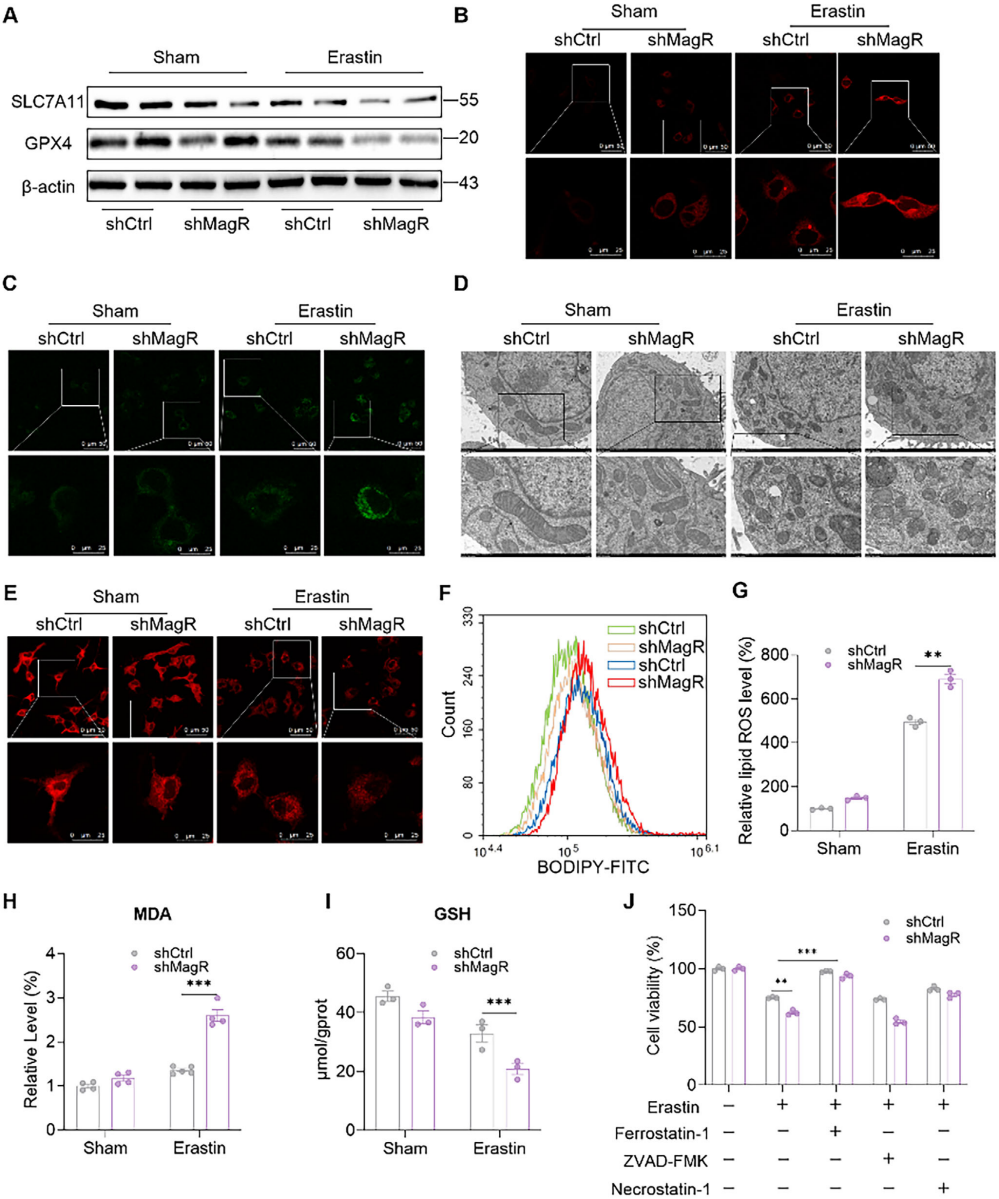
MagR deficiency enhanced erastin‐induced ferroptosis. Erastin (5 µm) was used to induce ferroptosis in negative control and MagR‐knockdown Huh7 cells. (A) The protein expression of GPX4 and SLC7A11 in negative control and MagR‐knockdown Huh7 cells following erastin treatment for 24 h. β‐actin was used as a loading control. (B) Representative FerroOrange fluorescence images. (C) Representative Mito‐FerroGreen fluorescence images. (D) Representative TEM images of mitochondria. (E) Representative MitoTracker fluorescence images. (F, G) Lipid ROS levels (F), with quantitative analysis (G). (H) Relative MDA level. (I) GSH level. (J) Cell viability after erastin treatment in the presence or absence of ferrostatin‐1(5 µM), Z‐VAD‐FMK (10 µM), or necrostatin‐1(5 µM). *n* = 3; data are presented as mean ± SD; **p < 0.01 and ***p < 0.001.

Elevated intracellular iron accumulation is a critical driver of ferroptosis. It facilitates subsequent lipid peroxidation via interconnected signaling cascades, which is a hallmark of this iron‐dependent cell death pathway [11]. Given our prior findings that MagR is involved in regulating cellular iron homeostasis, we next sought to investigate whether MagR directly modulates ferroptosis in HCC cells. To address this, we used erastin, a well‐established inducer of ferroptosis, to treat Huh7 cells, and compared the ferroptotic responses between MagR‐knockdown and control cells. First, we examined the expression of key ferroptosis‐related proteins: GPX4, a core suppressor of ferroptosis that neutralizes lipid peroxides, and SLC7A11, a cystine‐glutamate antiporter essential for GSH synthesis (a cofactor for GPX4). As shown in Figure 5A, erastin treatment significantly reduced GPX4 and SLC7A11 expression in control cells. Notably, this reduction was even more pronounced in MagR‐knockdown cells, suggesting a potential synergistic effect of MagR deficiency and erastin on ferroptosis induction. Ferroptosis is defined by two core biochemical features: increased labile Fe^2^^+^ levels and accumulation of lipid peroxides [11]. Consistent with this, after erastin treatment, MagR‐knockdown Huh7 cells exhibited a significantly higher intracellular Fe^2^^+^ level than control cells, as shown by FerroOrange staining (Figure 5B). Given MagR's mitochondrial localization, we further assessed the mitochondrial Fe^2^^+^ levels using the Mito‐FerroGreen probe. As illustrated in Figure 5C, MagR knockdown exacerbated the erastin‐induced Fe^2^^+^ accumulation in mitochondria, indicating that MagR may regulate ferroptosis, at least in part, by controlling mitochondrial iron levels.

Mitochondrial dysfunction and morphological alterations (e.g., reduced mitochondrial volume, disrupted cristae) are also characteristic of ferroptosis [11]. TEM analysis showed that erastin treatment alone caused reduced mitochondrial volume and increased cristae breakage in control cells. These pathological changes were markedly more severe in MagR‐knockdown cells (Figure 5D). Complementing the TEM data, MitoTracker fluorescence (a readout of mitochondrial function and quantity) was significantly decreased after erastin treatment, with a greater reduction observed in MagR‐knockdown cells (Figure 5E), confirming that MagR deficiency amplifies erastin‐induced mitochondrial impairment.

We next quantified lipid peroxide accumulation using two complementary approaches: C11‐BODIPY staining (flow cytometry) and MDA measurement (a stable end product of lipid peroxidation). As shown in Figure 5F,G, erastin treatment significantly increased the intracellular lipid ROS levels in control cells, and this increase was further enhanced by MagR knockdown. Similarly, erastin‐induced MDA elevation was more pronounced in MagR knockdown cells than in control cells (Figure 5H). Additionally, GSH depletion, a key event preceding ferroptosis, as it impairs GPX4 activity, was significantly accelerated in MagR‐knockdown cells after erastin treatment (Figure 5I).

To confirm that the enhanced cell death observed in MagR‐knockdown cells was specifically ferroptosis (rather than apoptosis or necrosis), we treated cells with pathway‐specific inhibitors: ferrostatin‐1 (ferroptosis inhibitor), Z‐VAD‐FMK (apoptosis inhibitor), or necrostatin‐1 (necroptosis inhibitor). As shown in Figure 5J, ferrostatin‐1 effectively reversed erastin‐induced cell death in both control and MagR‐knockdown cells. In contrast, Z‐VAD‐FMK and necrostatin‐1 had no significant effect on cell survival, ruling out apoptosis and necroptosis as the primary cell death mechanisms.

Collectively, these results demonstrate that MagR knockdown significantly potentiates erastin‐induced ferroptosis in HCC cells, likely by exacerbating iron accumulation (particularly in mitochondria), mitochondrial dysfunction, and lipid peroxidation, further establishing MagR as a key regulator of ferroptosis in HCC.

### 3.6 FTH1 Overexpression Attenuates the Increased Ferroptosis Sensitivity of MagR‐Knockdown Cells

**FIGURE 6:**
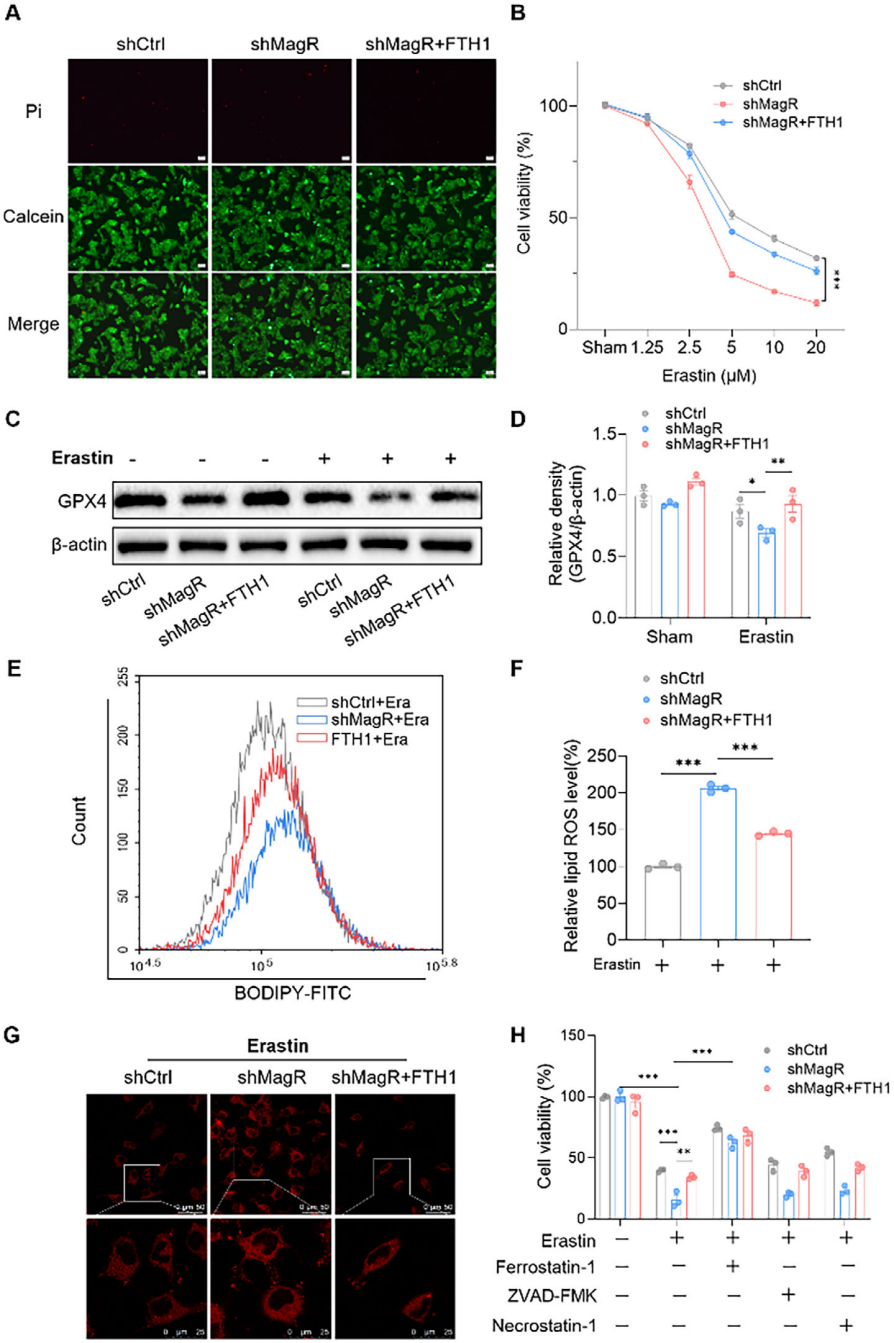
FTH1 overexpression enhanced ferroptosis resistance in the MagR‐knockdown cells. Erastin (5 µm) was used to induce ferroptosis in negative control, MagR‐knockdown, and FTH1‐ overexpression Huh7 cells. (A) Representative images of Calcein/PI fluorescence after erastin treatment for 24 h. (B) Cell viability after treatment with the indicated concentration of erastin for 24 h. (C, D) The protein expression of GPX4 (C), with the quantitative analysis (D). β‐actin was used as a loading control. (E, F) Lipid ROS level (E), with the quantitative analysis (F). (G) Representative FerroOrange fluorescence images. (H) The cell viability of negative control, MagR‐knockdown, and FTH1 overexpression Huh7 cells after erastin treatment in the presence or absence of ferrostatin‐1 (5 µM), Z‐VAD‐FMK (10 µM), or necrostatin‐1 (5 µM). *n* = 3; data are presented as mean ± SD; * *p* < 0.05, **p < 0.01 and ***p < 0.001.

Building on the aforementioned findings, we proposed the following hypothesis: Inhibition of MagR expression not only impairs mitochondrial function but also triggers an iron starvation response and drives the accumulation of labile Fe^2^^+^. To test this hypothesis, we overexpressed FTH1 in MagR‐knockdown Huh7 cells to restore iron balance, and then assessed whether this intervention could reverse the heightened ferroptosis sensitivity caused by MagR suppression. First, we evaluated cell death using PI/Calcein‐AM staining (a dual‐fluorescence assay that distinguishes dead cells [PI‐positive] from live cells [Calcein‐AM‐positive]). As shown in Figure 6A, MagR knockdown significantly increased erastin‐induced cell death, whereas FTH1 reconstitution markedly reduced this effect. The CCK‐8 assay yielded consistent results. MagR knockdown reduced cell viability under erastin treatment, while FTH1 overexpression markedly reversed viability to levels comparable to those of the control cells (Figure 6B). To dissect the mechanism underlying this rescue effect, we measured key ferroptosis‐related markers after erastin treatment. First, we examined GPX4, a critical ferroptosis suppressor whose reduction is tightly linked to ferroptosis induction. As illustrated in Figure 6C,D, erastin‐induced downregulation of GPX4 was significantly less pronounced in FTH1‐overexpressing/MagR‐knockdown cells than in MagR‐knockdown cells alone. Next, we assessed lipid ROS (a hallmark of ferroptosis) using C11‐BODIPY staining. FTH1 overexpression substantially reduced erastin‐induced lipid ROS accumulation (Figure 6E,F) and Fe^2^^+^ level (Figure 6G) in MagR‐knockdown cells, confirming that FTH1 overexpression effectively mitigates Fe^2^^+^ accumulation caused by MagR deficiency. To further validate that the rescued cell death was specifically ferroptosis, we treated cells with ferrostatin‐1 (a selective ferroptosis inhibitor). As shown in Figure 6H, ferrostatin‐1 reversed erastin‐induced cell death in all three groups (control, MagR‐knockdown, and MagR‐knockdown + FTH1‐overexpressing cells). This result not only confirms that the observed cell death was ferroptosis but also supports that FTH1 exerts its protective effect by targeting ferroptosis‐related pathways, consistent with its role in restoring iron homeostasis.

Together, these data demonstrate that MagR knockdown enhances the sensitivity of HCC cells to erastin‐induced ferroptosis by promoting Fe^2^^+^ accumulation. Critically, restoration of intracellular iron homeostasis through FTH1 overexpression eliminates this effect—providing direct evidence that MagR regulates ferroptosis sensitivity in HCC cells primarily through its control of iron metabolism.

### 3.7 Suppression of MagR Expression Enhances Sorafenib‐Induced Ferroptotic Cell Death

Sorafenib is a first‐line systemic therapeutic agent for patients with advanced HCC. However, the emergence of primary or acquired sorafenib resistance in HCC cells severely limits its clinical efficacy [41]. Given our previous findings that MagR regulates ferroptosis, a cell death pathway closely associated with cancer therapy response, we next sought to explore whether MagR modulates the sensitivity of HCC cells to sorafenib treatment. First, we compared the MagR expression between sorafenib‐resistant (SR) and sorafenib‐sensitive Huh7 cell lines. As shown in Figure S5A,B, MagR expression was significantly upregulated in SR Huh7 cells relative to their sensitive counterparts, hinting at a potential link between MagR overexpression and sorafenib resistance.

To investigate the role of MagR in sorafenib resistance in HCC, we knocked down MagR in SR Huh7 cells. The knockdown of MagR significantly improved the sensitivity of SR Huh7 cells to sorafenib compared to control SR Huh7 cells, as evidenced by the Calcein/PI and CCK‐8 assay results (Figure S5C,D). Meanwhile, the number of colony formation in MagR‐knockdown SR Huh7 cells was reduced than that in control SR Huh7 cells (Figure S5E).

**FIGURE 7:**
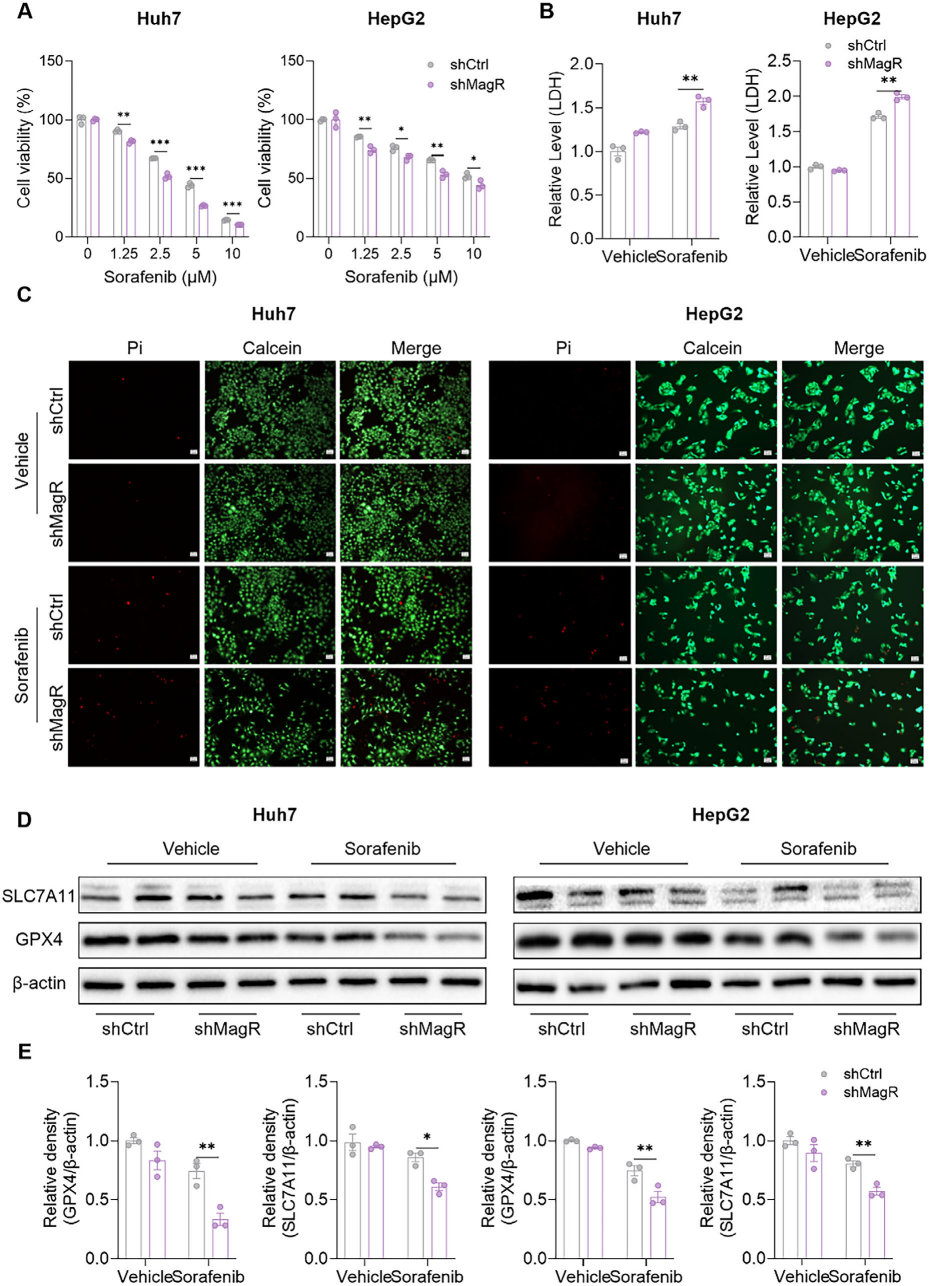
Suppression of MagR expression enhanced sorafenib‐induced ferroptotic cell death. Sorafenib (5 µM) was used to treat negative control and MagR‐knockdown Huh7 and HepG2 cells. (A) The cell viability of negative control and MagR‐knockdown HCC cells after different concentration sorafenib treatment for 24 h. (B) The LDH level in culture supernatants after sorafenib treatment for 24 h. (C) Representative images of Calcein/PI fluorescence. (D, E) The protein expression of GPX4 and SLC7A11 (D), with the quantitative analysis (E). β‐actin was used as a loading control. *n* = 3; data are presented as mean ± SD; * *p* < 0.05, **p < 0.01, and ***p < 0.001.

Furthermore, we treated MagR‐knockdown and control HCC cells with a gradient of sorafenib concentrations and assessed cell viability via the CCK‐8 assay. As illustrated in Figure 7A, sorafenib reduced HCC cell viability in a concentration‐dependent manner in both groups. Notably, the toxic effect of sorafenib was significantly enhanced in MagR‐knockdown cells compared to controls, and this difference was concentration‐dependent. When sorafenib reached a concentration of 5 µM, the disparity in cell viability between the two groups was most prominent. Thus, this concentration was selected for subsequent experiments because this concentration provided the largest dynamic range for evaluating MagR‐dependent sensitization to sorafenib. We then confirmed sorafenib‐induced cell damage by measuring LDH release (a marker of cell membrane integrity loss). As shown in Figure 7B, MagR deficiency exacerbated sorafenib‐induced LDH elevation, indicating more severe cell damage in MagR‐knockdown cells. Consistently, PI/Calcein‐AM staining showed that MagR knockdown significantly increased sorafenib‐induced HCC cell death compared with the control group (Figure 7C). The opposite results were observed in MagR‐overexpressing HCC cells (Figure S6).

Given our prior evidence that MagR regulates ferroptosis, we next explored whether MagR modulates sorafenib sensitivity by influencing ferroptotic pathways. We first examined the expression of two key ferroptosis suppressors: GPX4 and SLC7A11. As shown in Figure 7D,E, sorafenib treatment alone reduced GPX4 and SLC7A11 expression in control cells. Importantly, this reduction was significantly more pronounced in MagR‐knockdown cells, suggesting that MagR deficiency amplifies sorafenib‐induced ferroptosis signaling. To confirm that ferroptosis is the primary mechanism underlying the enhanced sorafenib sensitivity in MagR‐knockdown cells, we treated cells with pathway‐specific inhibitors: ferrostatin‐1 (ferroptosis inhibitor), Z‐VAD‐FMK (apoptosis inhibitor), or necrostatin‐1 (necroptosis inhibitor). As presented in Figure S7, ferrostatin‐1 effectively reversed sorafenib‐induced cell death in both Huh7 and HepG2 cells (regardless of MagR status). In contrast, Z‐VAD‐FMK and necrostatin‐1 had minimal impact on sorafenib‐induced cell death, ruling out apoptosis and necroptosis as the main contributors to the enhanced sensitivity observed in MagR‐knockdown cells.

Collectively, these findings demonstrate that MagR modulates the therapeutic efficacy of sorafenib in HCC cells, at least in part, by regulating ferroptosis. Specifically, MagR knockdown enhances sorafenib sensitivity by exacerbating ferroptotic signaling, whereas MagR upregulation (as seen in sorafenib‐resistant cells) may contribute to treatment resistance, highlighting MagR as a potential target to overcome sorafenib resistance in HCC.

### 3.8 Suppression of MagR Enhances the Anticancer Activity of Sorafenib In Vivo

To validate MagR's role in modulating sorafenib efficacy for HCC treatment in vivo, we established a subcutaneous xenograft model by injecting nude mice with MagR‐suppressed Huh7 cells (with or without recombinant FTH1 overexpression). Once tumors were established, the mice were treated with sorafenib. As shown in Figure S8, MagR knockdown significantly inhibited the in vivo tumor growth, whereas FTH1 overexpression partially reversed this effect, consisting with the in vitro findings. After 2 weeks of sorafenib treatment, subcutaneous tumors in all treated groups were significantly smaller than those in the untreated group, with the smallest tumor volume observed in the MagR‐knockdown + sorafenib group (Figure S8). These results confirm that MagR deficiency enhances the therapeutic efficacy of sorafenib against HCC in vivo.

Nanomedicine‐based strategies for HCC treatment have gained substantial attention, with remarkable progress in improving drug delivery efficiency and targeting specificity [42]. Sorafenib‐loaded nanoparticles (SOR‐NPs) offer advantages such as high bioavailability, active tumor targeting, and reduced off‐target effects, making them a promising approach for cancer therapy [43]. Building on this, we therefore constructed a novel multifunctional nanoparticle system (i.e., CC@siMagR&SOR@PP NPs), co‐loaded with MagR small interfering RNA and sorafenib (Supporting Information). We first performed a series of experiments to validate the successful synthesis, physicochemical properties, and biosafety of these nanoparticles (Figure S9).

TEM images revealed that CC@siMagR&SOR@PP NPs exhibited good dispersibility and a uniform spherical morphology (Figure S9A), a critical feature for stable in vivo delivery. Spectroscopic analysis showed overlapping characteristic peaks corresponding to both MagR‐siRNA and sorafenib, confirming the successful co‐loading of both therapeutic agents onto the nanoparticles (Figure S9B). After storage in DEPC water for 10 days, the hydrated dynamic particle size of the nanoparticles increased only slightly, indicating excellent colloidal stability, which is essential for preventing aggregation during storage and in vivo circulation (Figure S9C). The nanoparticles showed pH‐dependent release behavior: sorafenib was rapidly released under acidic conditions (pH 6.5, mimicking the tumor microenvironment and endosomal/lysosomal compartments), with a maximum release rate exceeding 90%. In contrast, release was significantly slower under physiological conditions (pH 7.4, mimicking normal tissues), with a maximum release rate of <40% even after 48 h (Figure S9D). This pH responsiveness ensures targeted drug release at the tumor site, minimizing systemic toxicity.

Functional assays further confirmed the nanoparticles’ therapeutic potential. A tube formation assay showed that CC@siMagR&SOR@PP NPs retained sorafenib's inherent anti‐angiogenic properties (Figure S9E), a key mechanism underlying sorafenib's anticancer effects. pH‐triggered drug release characteristics benefit the enhanced enrichment of siRNA and sorafenib in tumor tissues and improve the anti‐HCC effect of CC@siMagR&SOR@PP NPs. Therefore, we tested the cellular uptake under different incubation conditions. As shown in Figure S9F, we used Cy3‐labeled MagR‐siRNA to investigate the cellular uptake efficiency in Hepa1‐6 cells under different incubation conditions. The results showed that the fluorescence intensity of Hepa1‐6 cells incubated with CC@siMagR&SOR@PP NPs at pH 6.5 was comparable to that treated with the siRNA&Lipofectamine 2000 complex, whereas both the free MagR‐siRNA group and the pH 7.4 group treated with CC@siMagR&SOR@PP NPs exhibited relatively weak fluorescence intensity, indicating that CC@siMagR&SOR@PP NPs enhanced the cellular uptake of MagR‐siRNA in acidic environment and realized pH‐triggered targeted delivery. The nanoparticles effectively suppressed MagR expression in tumor tissues and exerted potent tumor cytotoxicity (Figure S9G–J).

Biosafety is a critical prerequisite for clinical translation. As shown in Figure S10A, compared with healthy mice, serum alanine aminotransferase (ALT), aspartate aminotransferase (AST), alkaline phosphatase (ALP), and total bilirubin (liver function indicators), as well as blood urea nitrogen and creatinine (renal function indicators) at different time points (1, 3, or 7 days after injection of CC@siMagR&SOR@PP NPs) had no significant difference with those of the control mice. Meanwhile, the body weight changes during the seven days showed that mice weight continued to increase steadily after injection of CC@siMagR&SOR@PP NPs, and there was no difference from the growth curve of control mice (Figure S10B). Short‐term (1 day) and long‐term (7 days) in vivo toxicity assessments showed that intravenous injection of CC@siMagR&SOR@PP NPs did not induce significant necrosis or inflammation in major organs (e.g., heart, liver, spleen, lung, kidney) of mice (Figure S10C). These results confirmed the nanoparticles’ favorable in vivo safety profile.

Precise release of therapeutic drugs in tumor tissues benefits the anti‐HCC effect. Therefore, we performed the in vivo tumor uptake assay to investigate whether the loaded drugs in CC@siMagR&SOR@PP NPs could be released accurately in the acidic microenvironment of tumors. Eight hours after mice were injected with CC@siMagR&SOR@PP NPs, strong fluorescence signals could be precisely observed in the tumor region of the liver, demonstrating the successful targeted delivery of siRNA to tumor regions (Figure S11A). For mice injected with the same amount of naked Cy5‐MagR‐siRNA, uniformly weak fluorescence signals distributed throughout the entire liver region, with no obvious difference in fluorescence intensity between the tumor region and normal liver tissue, indicating the rapid clearance of naked siRNA in vivo (Figure S11D). The ex vivo images of various organs and tumors further confirmed the targeted delivery and drug release of nanoparticles in liver tumors tissues (Figure S11B), whereas naked Cy5‐MagR‐siRNA was metabolized rapidly through the liver and kidneys (Figure S11E). Additionally, an enhanced Cy5 fluorescence signal was observed in the tumor site of the slices treated with CC@siMagR&SOR@PP NPs (Figure S11C), whereas the fluorescence signal mainly distributed at the normal liver tissue with almost no signal in the tumor region for Cy5‐MagR‐siRNA group (Figure S11F).

**FIGURE 8:**
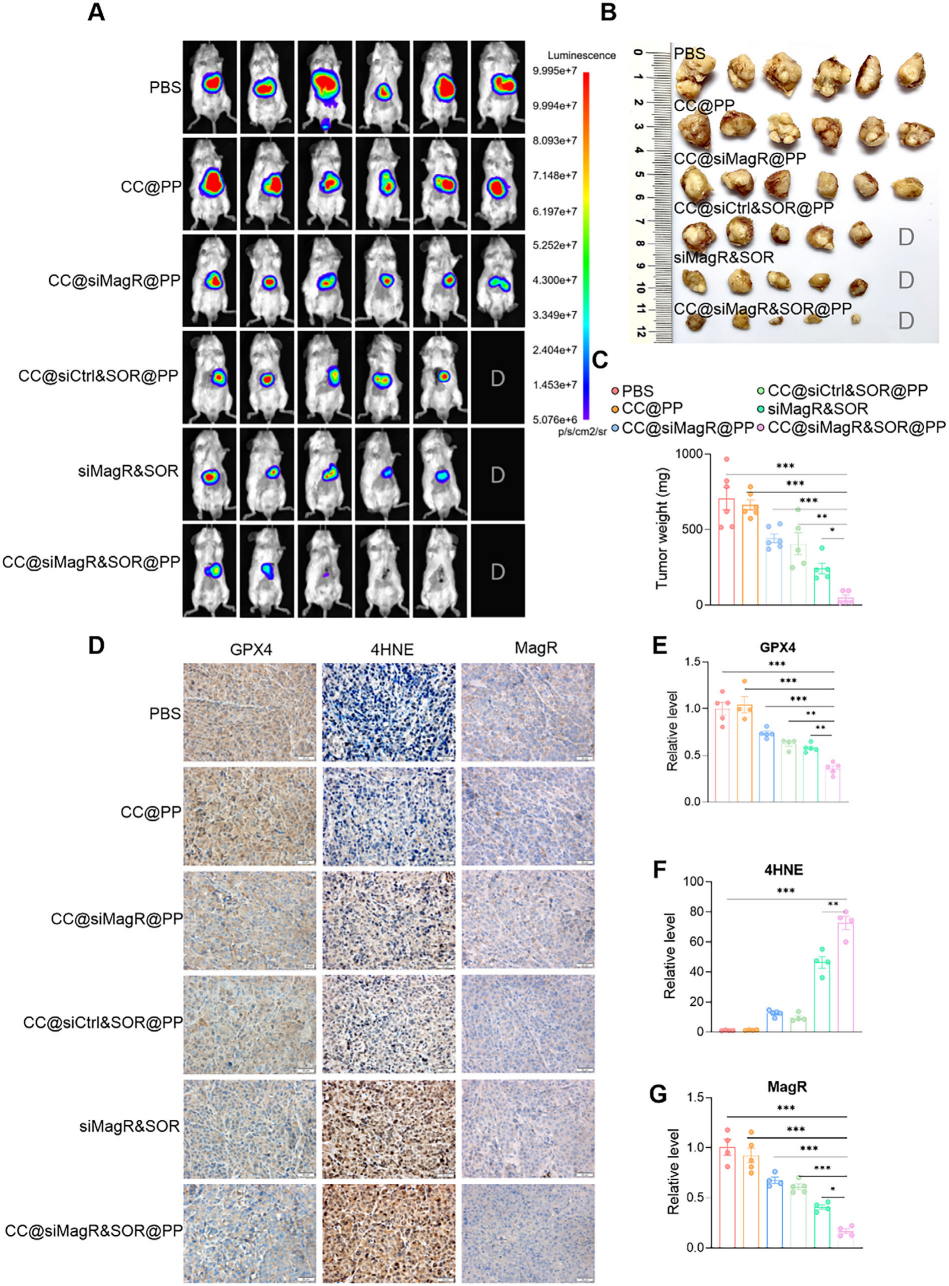
The CC@siMagR&SOR@PP NPs effectively inhibited mouse liver tumor growth in situ. The CC@siMagR&SOR@PP NPs and these control groups were used to treat mouse liver carcinoma in situ established by H22 cells for two weeks to evaluate their therapeutic effect. (A) In vivo real‐time visualization of tumors in the H22 orthotopic tumor‐bearing mouse at day 14 after the first injection. (B, C) Representative images of tumors collected on day 16 (B), with the quantitative analysis (C). (D–G) IHC analysis for GPX4, 4‐HNE, and MagR protein expression in indicated tumor specimens (D), with their quantitative analysis (E–G). *n* = 6; data are presented as mean ± SD; * *p* < 0.05, **p < 0.01, and ***p < 0.001.

To evaluate the therapeutic efficacy of CC@siMagR&SOR@PP NPs in a clinically relevant setting, we used them to treat a mouse orthotopic liver carcinoma model established with H22 cells. As shown in Figure 8A–C, compared with control groups (e.g., saline, free sorafenib, or single‐agent nanoparticles), CC@siMagR&SOR@PP NPs exhibited the strongest tumor inhibitory effect, validating the synergistic benefit of combining MagR silencing with sorafenib.

To explore the mechanism underlying this synergy, we performed IHC staining of tumor tissues for ferroptosis‐related markers: GPX4 and 4‐HNE (a stable end product of lipid peroxidation, a hallmark of ferroptosis). As shown in Figure 8D–G, all treatment groups showed decreased GPX4 expression and increased 4HNE levels, indicating ferroptosis induction. However, these changes were most pronounced in the CC@siMagR&SOR@PP NPs group. GPX4 downregulation was more significant, and 4HNE accumulation was more robust.

Collectively, these results demonstrate that co‐delivery of MagR‐siRNA and sorafenib via CC@siMagR&SOR@PP NPs significantly enhances ferroptosis induction in HCC cells, thereby synergistically improving the therapeutic efficacy of sorafenib against orthotopic liver tumors.

## 4 Discussion

In the current study, we identified MagR, also known as ISCA1, as a regulator of HCC progression and ferroptosis sensitivity. MagR was upregulated in HCC tissues and associated with adverse clinicopathological features and poor survival. Specifically, MagR was found to promote the proliferation, migration, and invasion of HCC cells by sustaining mitochondrial function and maintaining cellular iron homeostasis. Furthermore, we demonstrated that MagR deficiency enhances erastin‐ and sorafenib‐induced ferroptosis in HCC cells, which is mediated by the disruption of cellular iron homeostasis. Concurrently, we synthesized a pH‐responsive MagR‐siRNA and sorafenib‐loaded nanoparticle (CC@siMagR&SOR@PP NP) system, and experimental results confirmed its considerable antitumor potential. Notably, this work represents the first attempt to elucidate the previously unreported regulatory role of MagR in HCC progression. Additionally, it puts forward a novel perspective for optimizing sorafenib‐based therapeutic strategies and modulating ferroptosis in the context of HCC treatment.

Fe─S clusters (iron‐sulfur clusters, ISCs) are ancient protein cofactors required for apoprotein maturation, an essential process for fundamental biochemical reactions, including respiration, DNA replication, and heme biosynthesis. ISCs primarily exist in two major forms: [2Fe‐2S] and [4Fe‐4S] clusters [44]. The biosynthesis of [4Fe‐4S] clusters rely on a highly conserved and intricate biochemical pathway, which involves proteins such as NFU1, ISCA2, IBA57, BOLA3, and MagR (i.e., ISCA1) [45]. Among these proteins, MagR plays a more critical role in [4Fe‐4S] cluster formation. However, its biological functions in human diseases remain unclear. ISCA1 has been proposed as a prognostic biomarker in bladder carcinoma, clear cell renal cell carcinoma, and thyroid carcinoma [33, 34, 35]. In our research, bioanalytical results revealed that MagR expression was significantly higher in HCC tissues than in adjacent non‐tumor tissues. Moreover, high MagR expression was closely correlated with adverse clinicopathological characteristics and poor prognosis in patients with HCC. We then systematically investigated the impact of MagR expression on the malignant phenotypes (including proliferation, migration, and invasion) of HCC cell lines. Our findings indicated that MagR protein expression was associated with the proliferation, migration, and invasion capabilities of these HCC cell lines. Consistently, results from a xenograft mouse tumor model demonstrated that interfering with MagR expression could inhibit the growth of subcutaneous tumors in mice. Collectively, these results clearly confirm that MagR plays an essential role in sustaining the malignant properties of HCC cells.

Mitochondria are dynamic organelles that continuously undergo divide and fusion to maintain homeostasis [46]. To meet their high energy requirements, the mitochondrial function and dynamic behavior of HCC cells differ from those of normal cells. Studies have found that DRP1, a core molecule in mitochondrial fission, is significantly more highly expressed in HCC tissues than in adjacent non‐tumor tissues, and high DRP1 expression is closely associated with poor prognosis in HCC patients [47, 48, 49]. MFN2 regulates outer mitochondrial membrane fusion and has been linked to apoptosis and favorable prognosis in HCC [50, 51]. Enhanced mitochondrial biogenesis and oxidative phosphorylation have been shown to be key factors in promoting cancer metastasis [52]. PGC‐1α and TFAM are two proteins involved in mitochondrial biogenesis [53]. High PGC‐1α expression can enhance the invasion and migration of breast cancer, melanoma, and HCC [54], while TFAM knockdown promotes the metastasis of HCC to the lungs [55]. Given that MagR affects mitochondrial function through Fe‐S cluster assembly, any dysfunction or alteration in MagR expression may influence mitochondrial fission, fusion, and biogenesis. In our study, we found that DRP1, MFN2, PGC‐1α, and TFAM were significantly downregulated after MagR knockdown. Conversely, their protein expression was significantly upregulated after MagR overexpression. Although the expression trends of MFN2 and TFAM in our study differ from those in previous studies, we attribute this discrepancy to mitochondrial dynamic disorders caused by MagR deficiency.

Iron, an indispensable element for cellular proliferation and differentiation, maintains precise homeostatic regulation via complex storage and transport systems [56]. Among the numerous iron‐regulatory proteins, IRP1 and IRP2 (collectively referred to as IRPs) play the most critical roles in the iron starvation response [38]. Proteins involved in IRP regulation include TFR1 and divalent metal transporter 1(DMT1, linked to iron import), FTH1 (linked to iron storage), and ferroportin 1 (linked to iron export). This tripartite regulatory network ensures iron bioavailability for essential metabolic processes while preventing overload of the redox‐active labile iron pool [57]. One study demonstrated that inhibition of NFS1, a protein involved in ISC synthesis, can induce the iron starvation response in lung cancer cells and render these cells more susceptible to ferroptosis [58]. Similarly, another study found that frataxin (FXN) knockdown disrupts mitochondrial iron homeostasis, leading to cytosolic iron overload and mitochondrial dysfunction, which subsequently suppresses tumor growth [59]. MagR has also been shown to regulate the morphology of E. coli via intracellular iron accumulation [60]. In our study, MagR knockdown significantly upregulated IRP1, IRP2, and TFR1 both at mRNA and protein level, concomitant with an increase in total iron and intracellular Fe^2^^+^ concentration. Conversely, cells with MagR overexpression exhibited the opposite trend. These findings identify MagR as a critical nexus that integrates mitochondrial quality control with systemic iron metabolism by regulating Fe‐S cluster biogenesis and the stability of iron‐regulatory proteins.

Ferroptosis is an iron‐dependent form of regulated cell death characterized by lethal lipid ROS accumulation [11]. GSH, the primary cellular antioxidant, serves as an essential cofactor for GPX4, which is indispensable for eliminating lipid ROS [12]. The uptake of cystine (a key molecule in GSH synthesis) relies on SLC7A11. Regulation of SLC7A11 directly affects GSH levels and GPX4 activity, thereby controlling ferroptosis [61]. Thus, GPX4 and SLC7A11 are critical regulatory factors that inhibit ferroptosis, and their inhibition may partially trigger ferroptosis. Quantitative assessment of lipid ROS using the C11‐BODIPY (581/591) probe remains the gold standard for verifying ferroptosis [62]. MDA, a metabolite of lipid peroxides, can also serve as an indicator of ferroptosis [63]. Ferrous ions (Fe^2^^+^) play a pivotal role in ferroptosis, as they may promote the accumulation of lipid ROS by participating in the Fenton reaction [64]. Our results showed that MagR knockdown enhanced erastin‐induced ferroptosis, as evidenced by reduced GPX4 and SLC7A11 protein expression, decreased GSH levels, and increased Fe^2^^+^, MDA, and lipid ROS. Previous studies have demonstrated that HSPB1 [62], CISD1 [65], FXN [59], and HO‐1 [66] can regulate ferroptosis by modulating iron metabolism. Given MagR's impact on labile iron pool dynamics, we mechanistically defined its role in iron‐mediated ferroptosis regulation through FTH1 rescue experiments. Specifically, FTH1 reconstitution markedly restored intracellular iron homeostasis and abrogated the increased sensitivity to erastin‐induced ferroptosis in MagR‐knockdown cells. Collectively, these findings indicate that MagR regulates ferroptosis by influencing cellular iron homeostasis.

A 2013 study found that sorafenib can not only induce apoptosis and inhibit cell cycle progression in liver cancer cells but also trigger ferroptosis in these cells [23]. Since then, numerous studies have demonstrated that sorafenib‐induced ferroptosis can be regulated by several genes, including RB [67], p62 [68], MT‐1G [69], CISD1 [65], ceruloplasmin [70], and SLC27A5 [71]. However, the specific underlying mechanism remains to be elucidated. In our results, MagR knockdown significantly enhanced sorafenib toxicity in HCC cells. Additionally, MagR knockdown further exacerbated the sorafenib‐induced reduction in SLC7A11 and GPX4 expression. Therefore, we speculate that MagR may modulate sorafenib's toxicity in HCC cells by regulating ferroptosis.

Our research provides a theoretical basis for the development of gene therapy strategies based on targeted MagR regulation for the clinical treatment of HCC. However, conventional viral vectors have translational limitations, including limited targeting and specificity, low transfection efficiency, and potential immunotoxicity, making the direct application of gene‐targeted therapy still challenging [72]. Sorafenib‐loaded nanoparticles (SOR‐NPs) exhibit high drug delivery efficiency and bioavailability, and can actively target tumor tissues [73]. Additionally, SOR‐NPs have a smaller particle size and larger specific surface area, which improves the solubility of sorafenib [43]. By adjusting the properties of SOR‐NPs, drug delivery to target tumor tissues can be facilitated. In this study, building on SOR‐NPs, we developed a pH‐responsive polymeric nanotherapeutic agent (CC@siMagR&SOR@PP NPs) that co‐loads MagR‐siRNA and sorafenib. This nanosystem can specifically deliver MagR‐siRNA and sorafenib to liver tumor sites. This combined therapy can silence MagR expression in liver tumor cells while inhibiting liver tumor growth through its anti‐angiogenic effects and enhanced induction of ferroptosis. We anticipate that this novel nanoparticle‐based gene‐chemotherapy combination strategy will be clinically useful in the future and may also be applicable to other tumor types.

In the present study, there are several limitations that should be acknowledged. First, the number of clinical HCC tissue samples collected was small, and data on patients’ clinical etiologies and tumor characteristics were insufficient. Therefore, more samples and detailed data are needed to further validate MagR's role as an HCC tumor marker. Second, although we demonstrated that MagR affects the biological properties of HCC cells, the specific molecular mechanisms involved remain to be further explored. Finally, this study is the first to show that MagR can regulate ferroptosis by altering intracellular Fe^2^^+^ concentrations. However, whether MagR can also regulate ferroptosis by participating in other ferroptosis‐related pathways, such as the System Xc^−^/GPX4/GSH axis, remains to be investigated.

## 5 Conclusion

In summary, our study provides compelling evidence that MagR contributes to the carcinogenesis and progression of HCC by sustaining mitochondrial function and regulating cellular iron homeostasis. These findings support MagR as a novel prognostic biomarker and a promising potential therapeutic target for HCC. Additionally, we propose a novel therapeutic strategy that leverages sorafenib‐induced ferroptosis to improve HCC treatment outcomes.

## Author Contributions

**Rongqian Wu** and **Tao Wang** conceived the study and designed these experiments. **Chenghua Song**, Tao Wang and **Dan Li** synthesized the nanoparticles and conducted related experiments. Tao Wang, **Xin He**, **Mengzhou Wang**, **Wuming Liu** and **Xiaolin Wang** participated in the data collection and analysis. Xin He, **Yuanyuan Zhang**, **Jia Zhang** and **Lin Zhang** performed biological experiments. Chenghua Song and Tao Wang drafted the manuscript. **Zheng Wu**, **Yu Zhang**, **Yi Lyu** and Rongqian Wu revised the manuscript.

## Conflicts of Interest

The authors declare no conflicts of interest.

## Supporting information




**Supporting File 1**: advs78015‐sup‐0001‐SuppMat.docx.

**Supporting File 2**: advs78015‐sup‐0002‐SuppMat.docx.

## Data Availability

The data that support the findings of this study are available from the corresponding author upon reasonable request.
