# Supplementary material for: Magnetoreceptor Promotes Hepatocellular Carcinoma Development and Blunts Sorafenib‐Induced Ferroptosis by Regulating Iron Homeostasis
Source: Adv Sci (Weinh). 2026 Sep 27:e78015. Online ahead of print. doi: 10.1002/advs.78015 (PMC13616415; doi:10.1002/advs.78015)
Supplement: Supplementary file 1 [file ADVS-9999-e78015-s001.docx]

**SUPPLEMENTARY MATERIALS FOR**

**Magnetoreceptor Promotes Hepatocellular Carcinoma Development and Blunts Sorafenib-Induced Ferroptosis by Regulating Iron Homeostasis**

Tao Wang^1,3†^, Chenghua Song^1,2†^, Xin He^4†^, Mengzhou Wang^1,2^, Wuming Liu^1,2^, Xiaolin Wang^5^, Yuanyuan Zhang^6^, Jia Zhang^7^, Lin Zhang^1,2^, Dan Li^1^, Yu Zhang^3^, Zheng Wu^2^, Yi Lyu^1,2^, Rongqian Wu^1*^

^1^National Local Joint Engineering Research Center for Precision Surgery and Regenerative Medicine, Shaanxi Provincial Center for Regenerative Medicine and Surgical Engineering, The First Affiliated Hospital of Xi’an Jiaotong University, Xi’an, Shaanxi, China

^2^Department of Hepatobiliary Surgery, The First Affiliated Hospital of Xi’an Jiaotong University, Xi’an, Shaanxi, China

^3^Department of Hepatobiliary and Pancreas Surgery, Sichuan Provincial People’s Hospital, University of Electronic Science and Technology of China, Chengdu, Sichuan, China

^4^Department of Gastroenterology, The First Affiliated Hospital of Xi'an Jiaotong University, Xi'an, Shaanxi, China

^5^Department of Pathology, The First Affiliated Hospital of Xi'an Jiaotong University, Xi’an, Shaanxi, China

^6^Department of Pediatrics, The First Affiliated Hospital of Xi'an Jiaotong University, Xi’an, Shaanxi, China

^7^Department of Gastroenterology, The Second Affiliated Hospital of Xi'an Jiaotong University, Xi’an, Shaanxi, China

^†^These authors contributed equally to this work and share first authorship.

^*^Correspondence: Rongqian Wu, rwu001@mail.xjtu.edu.cn (ORCID ID: 0000-0003-0993-4531)

The materials and methods about the synthesis of CC@siMagR&SOR@PP NPs and their application in treating mouse liver carcinoma in situ were described as following：

**Materials and reagents**

Chloroform, methanol, paraformaldehyde was purchased from Sinopharm Chemical Reagent Co. Ltd. Sorafenib was purchased from Aladdin. mPEG5K-PAE10K (PP, average MW 15000) was obtained from Xi'an Ruixi Biological Technology Co, Ltd. Carboxymethyl chitosan (CMCS, M_W_ = 20,000–50000, carboxylation degrees: 87–90%) was purchased from Solarbio Life Science. D-Luciferin potassium salt, DEPC water and Hoechst 33,258 was purchased from Beyotime Biotechnology. Lipoamine-2000 was purchased from Invitrogen. Urea assay kit (BUN), alanine aminotransferase assay kit, alkaline phosphatase assay kit, aspartate aminotransferase assay kit, total bilirubin (T-BIL) kit and creatinine assay kit were purchased from Nanjing Jiancheng. CCK-8 was purchased from Dojindo. These siRNAs used in this experiment were obtained from GenePharma Co, Ltd.

**Mouse siRNA oligo sequence**

MagR-siRNA: sense: 5’-GGG CUG UGA GCA AGA GAA ATT-3’; antisense: 5’- UUU CUC UUG CUC ACA GCC CTT-3’.

Vector-siRNA: sense: 5’- UUC UCC GAA CGU GUC ACG UTT -3’; antisense: 5’- ACG UGA CAC GUU CGG AGA ATT -3’

**Cell culture**

The murine HCC cells Hepa1-6 (RRID: CVCL_0327), H22 (RRID: CVCL_H613), and Human umbilical vein endothelial cells (HUVECs, RRID: CVCL_2959) were purchased from Procell Life Science & Technology Co. Ltd. Luciferase labeled H22 cells (Luc-H22) were constructed by gene transfection with LV-LUC-PURO lentiviruses (HANBIO). Hepa1-6 cells were cultured in DMEM medium containing 10% FBS, 1% penicillin/streptomycin and 1% sodium pyruvate. HUVECs were cultured in MCDB131 medium supplemented with 5% FBS, growth factor additives and 1% penicillin/streptomycin. Luc-H22 cells were cultured in 1640 medium containing 10% FBS and 1% penicillin/streptomycin. All cell lines were authenticated by short tandem repeat (STR) profiling and tested negative for mycoplasma contamination before use. All cells were cultured in a cell culture incubator with 5% CO_2_ and a constant temperature of 37 ℃.

**Preparation of** **CC@siMagR&SOR@PP NPs**

We synthetized the CC@siMagR&SOR@PP NPs as previously described.

(1) 1 mg of sorafenib was dissolved in 1 mL of a chloroform/methanol (V: V = 1:1) mixture, and then the mixture was slowly added to 10 mL chloroform/methanol (V:V = 1:1) mixture containing 10 mg of mPEG5K-PAE10K under stirring.

(2) Gently stir the above mixture at room temperature for at least 1 h, then slowly drop it into 10 mL stirred DEPC water. Then, under the protection of an ice water bath, sonicate the mixture using a probe-type sonifier at 275 W for 4 min.

(3) Transfer the above reaction solution to a rotary evaporator and rotate at 37 ℃ for 1 h to remove the organic solvent. Then, the SOR@PP NPs dispersed in DEPC water were condensed by use ultrafiltration.

(4) Collect the filtrate and determine the residual amount of sorafenib in the filtrate by high-performance liquid chromatography.

(5) The drug loading efficiency (DLE) of sorafenib in SOR@PP NPs is calculated using the following formula:
DLE (wt.%) = (initial drug weight - residual drug weight) / mPEG5K-PAE10K weight

(6) Prepare a 20 µM Mag-siRNA solution with DEPC water, take 5 µL of the siRNA solution and mix it with different volumes of SOR@PP NPs solution (sorafenib concentration: 1 mg/mL), and then adjust the total volume to 100 µL with DEPC water.

(7) After vortexing the above mixture for at least 15 s, incubate it at room temperature for at least 30 min.

(8) The optimal ratio of Mag-siRNA to SOR@PP NPs was determine by agarose gel electrophoresis. Synthesize siMagR& SOR@PP NPs based on the optimal ratio.

(9) Slowly add different concentrations of CMCS to a fixed concentration of siMagR&SOR@PP NPs solution, vortex, and incubate at room temperature for at least 30 min.

(10) The optimal ratio of siRNA to CMCS was determined by agarose gel electrophoresis. Then synthesize CC@siMagR&SOR@PP NPs based on the optimal ratio.

**Characterizations of CC@siMagR&SOR@PP NPs**

(1) After negative staining of CC@siMagR&SOR@PP NPs with uranyl acetate, the morphology, size and distribution of the nanoparticles were observed under a transmission electron microscope.
(2) The ultraviolet-visible absorption spectra of CC@siMagR&SOR@PP NPs were measured using a UV-Vis spectrophotometer.
(3) The drug release rate of CC@siMagR&SOR@PP NPs under different pH conditions was analyzed by high performance liquid chromatography (HPLC).

**In vitro cellular uptake assay**

The cellular uptake assay was conducted on Hepa1-6 cells, which was transfected by Cy3-MagR-siRNA alone, Cy3-MagR-siRNA + Lipo2000, or CC@siMagR&SOR@PP NPs in a medium with a pH of 6.5 or 7.4. After incubation at 37 ℃ for 6 h, the fluorescence density of these cells was monitored with a fluorescence microscope.

**In vitro MagR interference assay**

The MagR interference assay was also performed on Hepa1-6 cells. After transfecting Cy3-MagR-siRNA + Lipo2000, or CC@siMagR&SOR@PP NPs into Hepa1-6 cells in a medium at pH 6.5 for 72 h, the protein of Hepa1-6 cells were extracted and the expression of MagR protein was detected by Western blot.

**In vitro cytotoxicity assay**

The cytotoxicity of CC@siMagR&SOR@PP NPs was examined using CCK-8 assay kit. Briefly, the H22 cells were treated with different concentrations of CC@siMagR&SOR@PP NPs (with the MagR-siRNA concentrations were 0, 0.125, 0.25, 0.5, 0.75, 1.25, and 2.5 µg/ml) for 24 h or 48 h. Then 10 µL CCK-8 reagents were added for further incubation at 37 ℃ for 2 h. Absorbance was measured at 450 nm using a microplate reader.

**In vivo toxicity assay**

Twenty male Balb/c mice weighing ~25 g were administered intravenously with CC@siMagR&SOR@PP NPs or PBS (5 mice per group, MagR-siRNA 0.5 mg/mL, sorafenib 2.0 mg/mL, PP 20 mg/mL, and CMCS 0.5 mg/mL). The weights of mice were recorded continuously and the mice were sacrificed at 1, 3 or 7 days after administration. Mice blood samples were collected from the mice’s eyeballs for hepatic and renal function analyses. The lungs, hearts, kidneys, spleens and livers were harvested for fixation in 4% paraformaldehyde solution. The hematoxylin&eosin staining was carried out following the standard sample operation procedure by Y&K Bio (Xi’an, China). All the images were obtained from a Pannoramic DESK Slide Scanner (3D HISTECH, Hungary).

**Tube formation assay**

Human umbilical vein endothelial cells (HUVECs) were used in the tube formation experiment. Briefly, 2×10^4^ HUVECs cultured in 100 µL MCDB131 medium (pH 6.5) containing 5 μg/mL of sorafenib, CC@siMagR&SOR@PP NPs or equivalent CC@PP NPs were added to 96-well plate, which was preprocessed with 50 µL of the matrix gel. After incubation for 24 h, the cells were fixed with 4% paraformaldehyde. Micro-tubes formed were photographed using the Optical microscope.

**Mouse orthotopic tumor model**

The orthotopic tumor model of HCC was established as follows: Male Balb/c mice (5–6 week) were anesthetized with 2% isoflurane and 2 L/min oxygen flow during the whole surgical procedure. After exposing the median liver lobe through a 1-2cm in the midline abdominal skin, 1x10^6^ of Luc-H22 cells dissolved in 50 μL of PBS were slowly injected into the liver using a 1 mL syringe. Then the abdominal cavity was sutured, and the mice were placed on a warming pad for palinesthesia. Tumor growth was observed by small animal imaging apparatus or laparotomy.

**In vivo tumor uptake assay**

Eight days after the mouse orthotopic tumor model was prepared, tumor-bearing mice were administered intravenously with 200 μL of Cy5-MagR-siRNA or CC@siMagR&SOR@PP NPs (siRNA concentration: ~ 0.25 mg/mL). Bioluminescence images (10 min after Intraperitoneal injection with 200 μL of D-Luciferin potassium salt) and fluorescence images (emission and excitation wavelength: 640/680 nm) were obtained 8 h post injection using the Xenogen IVIS SPECTRUM small animal imaging system. Then, major organs including lung, heart, kidneys, liver, spleen and tumor was collected and embed with optimal cutting temperature compound (OCT) for further staining. Fluorescence images of the slices were obtained from a PannoramicMIDI Slide Scanner (3D HISTECH, Hungary).

**In vivo anticancer** **assay**

One week after H22 injection, the mice with similar tumor size were used in further experiments. These mice were randomly divided into 6 groups (6 mice per group), namely PBS, CC@PP, siMagR&SOR, CC@siMagR@PP, CC@siCtrl&SOR@PP and CC@siMagR&SOR@PP NPs groups (MagR-siRNA 0.5 mg/mL, sorafenib 2.0 mg/mL, PP 20 mg/mL, and CMCS 0.5 mg/mL). 100 μL of different drugs were administered intravenously at day 1, 4, 7, 10 and 13 with dosage of 2.5 mg/kg siRNA and 10 mg/kg sorafenib. One day after the drug injection was completed, D-luciferin potassium salt solution was injected intraperitoneally according to the instructions. The tumor growth was observed using a small animal imaging system. Three days after the last drug injection, the mice were sacrificed, and liver tissues were obtained for measurement and then embedded in paraffin for further experiments.
