# Supplementary material for: Magnetoreceptor Promotes Hepatocellular Carcinoma Development and Blunts Sorafenib‐Induced Ferroptosis by Regulating Iron Homeostasis
Source: Adv Sci (Weinh). 2026 Sep 27:e78015. Online ahead of print. doi: 10.1002/advs.78015 (PMC13616415; doi:10.1002/advs.78015)
Supplement: Supplementary file 2 [file ADVS-9999-e78015-s002.docx]

**SUPPLEMENTARY MATERIALS FOR**

**Magnetoreceptor Promotes Hepatocellular Carcinoma Development and Blunts Sorafenib-Induced Ferroptosis by Regulating Iron Homeostasis**

Tao Wang^1,3†^, Chenghua Song^1,2†^, Xin He^4†^, Mengzhou Wang^1,2^, Wuming Liu^1,2^, Xiaolin Wang^5^, Yuanyuan Zhang^6^, Jia Zhang^7^, Lin Zhang^1,2^, Dan Li^1^, Yu Zhang^3^, Zheng Wu^2^, Yi Lyu^1,2^, Rongqian Wu^1*^

^1^National Local Joint Engineering Research Center for Precision Surgery and Regenerative Medicine, Shaanxi Provincial Center for Regenerative Medicine and Surgical Engineering, The First Affiliated Hospital of Xi’an Jiaotong University, Xi’an, Shaanxi, China

^2^Department of Hepatobiliary Surgery, The First Affiliated Hospital of Xi’an Jiaotong University, Xi’an, Shaanxi, China

^3^Department of Hepatobiliary and Pancreas Surgery, Sichuan Provincial People’s Hospital, University of Electronic Science and Technology of China, Chengdu, Sichuan, China

^4^Department of Gastroenterology, The First Affiliated Hospital of Xi'an Jiaotong University, Xi'an, Shaanxi, China

^5^Department of Pathology, The First Affiliated Hospital of Xi'an Jiaotong University, Xi’an, Shaanxi, China

^6^Department of Pediatrics, The First Affiliated Hospital of Xi'an Jiaotong University, Xi’an, Shaanxi, China

^7^Department of Gastroenterology, The Second Affiliated Hospital of Xi'an Jiaotong University, Xi’an, Shaanxi, China

^†^These authors contributed equally to this work and share first authorship.

^*^Correspondence: Rongqian Wu, rwu001@mail.xjtu.edu.cn (ORCID ID: 0000-0003-0993-4531)

**Figure S1. High MagR expression indicated poor prognosis.**

(A) Relationship between MagR expression and clinicopathological features in HCC, including TNM-stage, tumor stage. (B) Kaplan Meier curves for overall survival, disease-specific survival and progress free interval. Data are presented as mean ± SD; **p* < 0.05 and ****p* < 0.001.

**Figure S2. MagR overexpression promoted proliferation, migration, and invasion of HCC cells.**

All these experiments were conducted on Huh7 and HepG2 cells. (A-D) the protein and mRNA expression of MagR in HCC cells transfected with negative control or MagR overexpression constructs (NC and MagR OE). (E) The proliferative ability between negative control or MagR-overexpression HCC cells. (F, G) Representative images of the colony formation(F), with the quantitative analysis (G). (H, I) Representative images of wound-healing (H), with the quantitative analysis (I). (J-L) Representative images of migration and invasion (J), with the quantitative analysis (K, L). n = 3; Data are presented as mean ± SD; **p* < 0.05, ***p* < 0.01, and ****p* < 0.001.

**Figure S3.** **MagR knockdown increases mitochondrial ROS and decreases mitochondrial membrane potential in HCC cells.**

All these experiments were conducted on Huh7 and HepG2 cells. (A, B) Representative images of MitoSOX fluorescence in negative control or MagR-knockdown HCC cells (A), with the quantitative analysis (B). (C, D) Representative images of JC-1 fluorescence. (E, F) the mRNA level of IRP1, IRP2, TFR1, FTL and FTH1. (G, H) The mRNA level of IRP1, IRP2, TFR1 FTL and FTH1. n = 3; Data are presented as mean ± SD; **p* < 0.05 and ***p* < 0.01.

**Figure S4. Overexpression of MagR enhanced the function of mitochondria and relieved the accumulation of free iron in HCC cells.**

All these experiments were conducted on Huh7 and HepG2 cells. (A, B) the protein expression of PGC-1α, DRP1, MFN2 and TFAM in negative control or MagR-overexpression HCC cells (A), with the quantitative analysis (B). β-actin was used as a loading control. (C, D) Representative Mitotracker fluorescence images (C), with the quantitative analysis (D). (E) ATP level in negative control or MagR-overexpression HCC cells. (F, G) the protein expression of IRP1, IRP2, TFR1, FTL and FTH1 (F), with the quantitative analysis(G). β-actin was used as a loading control. (H) Representative FerroOrange fluorescence images. (I) Total iron level. (J) Fe^2+^/Fe^3+^ ratio. n = 3; Data are presented as mean ± SD; **p* < 0.05, ***p* < 0.01 and ****p* < 0.001.

**Figure S5. MagR deficiency improved the sensitivity of** **sorafenib-resistant cells to sorafenib.**

(A) The cell viability of sorafenib-sensitive and sorafenib-resistant (SR) Huh7 cells after different concentration sorafenib treatment for 24 h. IC_50_: half-maximal inhibitory concentration. (B) The protein expression of ISCA1 in sorafenib-sensitive and SR Huh7 cells. β-actin was used as a loading control. (C) Representative images of Calcein/PI fluorescence of indicated groups following sorafenib (5 μM) treatment for 24 h. (D)Cell viability. (E) Representative images of the colony formation. n = 3; Data are presented as mean ± SD; ****p* < 0.001.

**Figure S6. MagR overexpression attenuates sorafenib-induced ferroptotic cell death.**

Sorafenib (5 μM) was used to treat negative control and MagR-overexpression Huh7 and HepG2 cells. (A) The cell viability of negative control and MagR overexpression HCC cells after different concentration sorafenib treatment for 24 h. (B) The LDH level in culture supernatants after sorafenib treatment for 24 h. (C) Representative images of Calcein/PI fluorescence. n = 3; Data are presented as mean ± SD; * *p* < 0.05 and ***p* < 0.01.

**Figure S7. MagR knockdown increased sorafenib-induced ferroptotic cell death of HCC cells.**

(A) The cell viability of negative control and MagR-knockdown Huh7 cells after sorafenib treatment for 24 h, in the presence or absence of ferrostatin-1(5 μM), Z-VAD-FMK (10 μM) or necrostatin-1(5 μM). (B) The cell viability of negative control and MagR knockdown HepG2 cells after sorafenib treatment for 24 h, in the presence or absence of ferrostatin-1(5 μM), Z-VAD-FMK (10 μM) or necrostatin-1(5 μM). n = 3; Data are presented as mean ± SD; **p < 0.01 and ***p < 0.001.

**Figure S8. MagR knockdown increased the anti-tumor efficiency of sorafenib in vivo.**

Liver tumor of mice was constructed by subcutaneous injection of Huh7 cells into the flank of nude mice. Nude mice were administrated with sorafenib for 2 weeks (10 mg/kg, intraperitoneally, once every other day). (A, B) The tumor volumes were measured every 3 days (A), with the representative tumor images at the 21^st^ day (B). n = 4; Data are presented as mean ± SD; **p* < 0.05 and ****p* < 0.001.

**Figure S9. The successful synthesis of CC@siMagR&SOR@PP NPs.**

(A) TEM image of CC@siMagR&SOR@PP NPs. (B) UV–Vis absorbance spectra of CC@siMagR&SOR@PP NPs and equivalent of SOR@PP NPs, MagR-siRNA, CMCS, PP NPs and sorafenib. (C) The hydrodynamic diameters of CC@siMagR&SOR@PP NPs at day 0 and day 10, stored in DNA/RNA free sterile water at 4 ℃. (D) In vitro sorafenib release curves from CC@siMagR&SOR@PP NPs over time in PBS buffer at pH 7.4 and pH 6.5. (E) The tube formation assay of HUVECs after treated with CC@siMagR&SOR@PP NPs and equivalent of CC@PP NPs, MagR-siRNA and sorafenib. (F) In vitro cellular uptake assay of Hepa 1–6 cells treated with, MagR-siRNA or CC@siMagR&SOR@PP NPs in different pH conditions. (G, H) In vitro MagR Interference Assay. The expression of MagR in Hepa1-6 cells treated with different nanoparticles or siRNA(G). with the quantitative analysis (H). (I, J) The cell viability of H22 cells with different treatments for 24 h (I) and 48 h (J). n = 3; Data are presented as mean ± SD; ***p* < 0.01 and ****p* < 0.001.

**Figure S10. In vivo cytotoxicity assay.** **CC@siMagR&SOR@PP NPs have no observable tissue damage.**

(A) Hepatic (ALT, AST, ALP and T-BIL,) and Renal (BUN and creatinine) function test. (B) Body weight changes in mice over seven days after being injected with CC@siMagR&SOR@PP NPs (n = 5). (C) Representative H&E staining images of different organs after treated mice with PBS or CC@siMagR&SOR@PP NPs for 1, 3 and 7 days. No observable tissue damage, necrosis and inflammation were observed. Scale bar: 200 μm.

**Figure S11. In vivo tumor uptake assay. CC@siMagR&SOR@PP NPs enhance the tumor’s uptake of siRNA.**

(A, D) Bioluminescence, brightfield and Cy5 fluorescence images obtained 8 h after the mice were injected with CC@siMagR&SOR@PP NPs (A) or free MagR-siRNA (D). (B, E) Ex vivo merge images of tumors and organs with CC@siMagR&SOR@PP NPs (B) and free MagR-siRNA (E). (C, F) Tumor slices images at 8 h after intravenous injection of different formulas. CC@siMagR&SOR@PP NPs (C) and free MagR-siRNA (F).
